# Residential Environments for Older Persons: A Comprehensive Literature Review (2005–2022)

**DOI:** 10.1177/19375867231152611

**Published:** 2023-04-19

**Authors:** Stephen Verderber, Umi Koyabashi, Catherine Dela Cruz, Aseel Sadat, Diana C. Anderson

**Affiliations:** 1 Centre for Design + Health Innovation, John H. Daniels Faculty of Architecture, Landscape and Design, University of Toronto, Ontario, Canada; 2 Institute for Health Policy, Management and Evaluation, Dalla Lana School of Public Health, University of Toronto, Ontario, Canada; 3 John H. Daniels Faculty of Architecture, Landscape and Design, University of Toronto, Ontario, Canada; 4 Boston University School of Medicine, MA, USA; 5 Jacobs, Dallas, TX, USA

**Keywords:** literature review, older persons, residential built environment, infection control, COVID-19, evidence-based design, best practices, nature engagement, occupant satisfaction, performance, well-being, health status, voluntary and involuntary relocation, future trends

## Abstract

**Background:**

Independent noninstitutional and institutional residential long-term care environments for older persons have been the subject of significant empirical and qualitative research in the 2005–2022 period. A comprehensive review of this literature is reported, summarizing recent advancements in this rapidly expanding body of knowledge.

**Purpose and Aim:**

This comprehensive review conceptually structures the recent literature on environment and aging to provide conceptual clarity and identify current and future trends.

**Method and Result:**

Each source reviewed was classified as one of the five types—opinion piece/essay, cross-sectional empirical investigation, nonrandomized comparative investigation, randomized study, and policy review essay—within eight content categories: community-based aging in place; residentialism; nature, landscape, and biophilia; dementia special care units; voluntary/involuntary relocation; infection control/COVID-19, safety/environmental stress; ecological and cost-effective best practices; and recent design trends and prognostications.

**Conclusions:**

Among the findings embodied in the 204 literature sources reviewed, all-private room long-term care residential units are generally safer and provide greater privacy and personal autonomy to residents, the deleterious impacts of involuntary relocation continue, family engagement in policy making and daily routines has increased, multigenerational independent living alternatives are increasing, the therapeutic role of nature and landscape is increasingly well-documented, ecological sustainability has increased in priority, and infection control measures are of high priority in the wake of the coronavirus pandemic. Discussion of the results of this comprehensive review sets the stage for further research and design advancements on this subject in light of the rapid aging of societies around the globe.

## Introduction

Environmental design research has evolved significantly over the past 50 years to be recognized as a distinct discipline centered on the transactional relationship between the built environment, design, and the improvement of the human condition. In the past quarter century, a subdiscipline of evidence-based research and design has focused on health and the built environment, addressing the spectrum of healthcare building types including hospitals, hospices, long-term care facilities, pediatric facilities, psychiatric and substance abuse treatment centers, and community-based outpatient clinics (Verderber, 2010; Verderber & Fine, 2000). The published literature has become increasingly complex, multifaceted and somewhat bifurcated with respect to the transactional role of the built environment and its impacts on older persons—especially in the context of the coronavirus pandemic. At this time, it is appropriate to take stock of the recent literature which addresses the broad range of residential built environments for older persons, their caregivers, and the families of older persons. A review of recent peer-reviewed quantitative and qualitative investigations and theoretical essays on noninstitutional residential settings (NIRS) and residential long-term care (RLTC) built environments can yield insight—particularly now—as societies around the world are experiencing the rapid aging of their populations while endeavoring to control and eradicate the virulent COVID-19 pandemic, which has had a disproportionally adverse impact on RLTC settings. The former settings consist of independent dwellings and congregate independent living housing. The latter housing types consist of assisted living facilities, continuing care retirement communities (CCRCs) with a skilled nursing component, and skilled nursing-only long-term care institutions. As of late 2022, 6.4 million persons have died globally from the coronavirus, and of this number, nearly 75% were over the age of 65 (World Health Organization, 2022). This review attempts to answer two broad research questions: First, “What significant trends are discernable in the recent research on the role of the built environment with respect to older persons residing in NIRS and RLTC settings?” Second, “How has the coronavirus pandemic impacted the design and occupancy of these two types of settings for older persons, and what recommended design interventions have emerged as a direct result?”

This review is centered on current knowledge, theoretical perspectives, and best practices, premised on the assumption this information is beneficial to a broad audience of architects, landscape architects, interior designers, administrators, direct care providers, and the families of older persons who reside in these settings. It consists of peer-reviewed research investigations, pertinent theoretical essays, and prognostications for the future. The aim of this evidence-based review is to inform design. The focus is on adverse medical outcomes, infection control and safety protocols, the growing role of residents’ families in the caregiving experience, salient person–nature/landscape transactions, ecological best practices, and caregiver job performance and satisfaction.

***This review is centered on current knowledge, theoretical perspectives, and best practices, premised on the assumption this information is beneficial to a broad audience of architects, landscape architects, interior designers, administrators, direct care providers, and the families of older persons who reside in these settings. It consists of peer-reviewed research investigations, pertinent theoretical essays, and prognostications for the future***.

## Method

The methodology consisted of a broad review of published, peer-reviewed quantitative and qualitative investigations and essays. The first step consisted of a key-word search to identify potentially relevant peer-reviewed publications. Forty-five key words were used, referring to NIRS and RLTC resident and staff outcomes, that is, wayfinding behavior, engagement with nature and landscape, infection control and COVID-19, medical errors, pain, stress, sleep patterns, privacy, personal autonomy, room personalization, and voluntary and involuntary relocation impacts. Second, referring to physical environment factors, that is, residential unit design and configuration; special care units for persons with dementia and related cognitive disorders; staff workstation design; daylighting, noise; and site amenities including exterior balconies, courtyards, greenhouses, and gardens. Third, related issues, that is, staff productivity, stress, family-centered care, noise mitigation, nature, views, landscape, nature representations, resident safety, satisfaction, well-being, and mortality, the future of NIRS and RLTC built environments, theoretical prognostications, and anticipated architectural and landscape design trends. Fourth, referring to facility infrastructure, that is, carbon neutral NIRS and RLTC facilities, sustainable design and operations, nontoxic materials, and renovation and retrofitting best practices.

A protocol established by Verderber et al. (2021) with respect to a comprehensive review of the literature on hospital-based intensive care built environments guided a set of extensive cross-searches using combinations of key words and phrases through the JSTOR and Google Scholar databases and further searches combing multiple databases including EBSCO, ScienceDirect, PsychINFO, MEDLINE, Ovid, ProQuest, PubMed, Web of Science, Science Digest, and NIH Public Access. This search process identified peer-reviewed studies or articles that directly referred to NIRS and/or RLTC healthcare physical built environments in the title or abstract, published between January 2005 and October 2022. The decision was made at the outset to include both empirical and qualitative peer-reviewed research investigations, as well as relevant peer-reviewed theoretical and opinion essays in order to broadly capture the scope, depth, and nuance of a rapidly evolving global subject. The initial search phase yielded 487 in-range sources, subsequently reduced in a second stage assessment to 257 peer-reviewed general primary sources. This pared down set of published sources met or exceeded the review team’s baseline benchmark for rigor and general thematic focus. In a third-stage assessment, these sources were further examined and reduced to a compendium of 204 core sources, reported below.

The research team carefully screened three types of peer-reviewed literature sources: (1) empirically based studies that examine the role and impact of the built environment or natural environment on resident, staff, and/or family outcomes; (2) qualitative studies that examine these same relationships; and (3) theoretical essays that examine the relationship between nursing best practices and administrative policies, and the planning and design of 24/7 NIRS and RLTC residential environments for older persons. Non-peer-reviewed white papers, research reports, minimum standards guidelines publications, and books on this subject were eliminated in the first wave of this screening process. This core compendium of literature sources was then interpreted by the research team with respect to how best to categorize this considerable body of knowledge in relation to the two aforementioned research questions.

This question arose: “How best to thematically structure this large body of information?” Eight thematic content categories were subsequently identified vis-à-vis an iterative, interpretative screening process. A number of themes emerged, beginning with the importance of addressing the fundamentals of older persons being able to reside in a noninstitutional, home-like residential setting in the community as well as the importance of providing 24/7 RLTC settings that are as home-like, dignified, and self-empowering as possible, referred to here as *residentialism*, combined with the importance of providing direct connections with the exterior realm (nature and landscape) both within the facility and in its immediate site and campus contexts. Next, a sizable literature had emerged on the planning, design, and impact of specialized RLTC care settings for persons with diminished cognitive abilities (dementia special care units). Next, a relatively small but thematically related, identifiable literature had appeared on the topic of voluntary and involuntary transferring from a noninstitutional to a 24/7 institutional care setting (voluntary as well as involuntary relocation). Next, a sizable literature had emerged on the deleterious impacts of infectious disease (including COVID-19) and the adverse health status and well-being impacts of environmental sources of stress on building occupants (infection control, safety). Next, a sizable literature was discerned on efforts to reduce the carbon footprints of RLTC settings for older persons and also recent facility management best practices (ecological and related facility management). Lastly, a sizable literature had emerged consisting of theoretical essays on the present and future of residential care settings for older persons (trends and prognostications).

In response to the aforementioned identifiable trends and priorities implicitly expressed in the literature, this compendium of literature sources was subsequently structured into the following eight thematic content categories: (1) Community-based non-RLTC Settings; (2) Residentialism; (3) Nature and Landscape; (4) Dementia Special Care Units (SCUs); (5) Voluntary/Involuntary Relocation; (6) Infection Control/COVID-19/Environmental Stress; (7) Sustainability/Facility Management; and (8) Design Trends/Prognostications. As such, these eight themes and their respective subthemes are not to be construed as a typology per se. In point of fact, collectively, this conceptualization is a set of aggregated themes. The results of this process are reported in Table 1, which describes each individual study or essay, as well as its research design/focus, the physical setting and sample population, key physical environment attributes addressed, outcome measures of health status and well-being, and lastly, the impact of NIRS and RLTC settings on health status and well-being outcomes and, where cited, behavioral and/or design-based recommendations. These thematic categories are reported below, with the sources variously cited within each category correspondingly populating (Table 1).

**Table 1.** Summary of Noninstitutional Residential Settings and Residential Long-Term Care (RLTC) Settings Comprehensive Literature Review (2005–2022)—Content Areas 1–8.

Citation	Research Design, Setting, and Sample	Built-Environment Attributes	Outcome Measures of Well-Being	Built Environment Impact on Outcome(s)
**(1) Community-based non-RLTC settings**				
(1a) Immediate neighborhood and urban environment				
Alley et al. (2007)	Empirical study: multisite; Canada and United States. Home-based community care: residents aged 65 and older	Transportation, housing options, and healthcare clinics	Role of social workers in facilitating access to physical resources	Accessible physical infrastructural amenities, that is, transit, healthcare, promote residents’ satisfaction
Aung et al. (2021)	Cross-sectional study: multisite; Japan. Home-based mail-in survey; 243 respondents, average age: 74 years	Pedestrian paths, access to civic spaces and buildings, and public transit	Environment-based factors that promote social network diversity, active aging, and quality of life	Municipalities need to uniquely prioritize greater elder access to community infrastructure resources
Baldwin et al. (2013)	Research review/policy. General, international.	Age-friendly architectural and civic resources	Satisfaction, health status	Social and cultural engagement preferred: access to resources: local shops, pubs, cafés, libraries, and parks
Buffel et al. (2012)	Multimethod policy review; United Kingdom and Belgium. Community based. Determinates of age-friendly cities	“Community Action in Later Life—Manchester Engagement,” in United Kingdom, and “Belgian Ageing Studies” projects	Identification of “good” and “optimal” aging in place urban attributes	The aged remain excluded in most urban communities. Policies needed to involve them in social, economic life.
Campbell (2015)	Cross-sectional observational study. Multisite; United States. Aged residents’ apartments	Social/communal spaces in local community	Travel/movement patterns; proximity to home	Psychosocial needs of community-based residents are important in designing social/communal spaces.
Forsyth et al. (2019)	Systematic narrative review. Multisite; United Sates. The aged 55 and over living alone.	Aging-in-place housing attributes	Predictors of isolation and unmet physical and social needs as function of housing type	Single-person households most susceptible to isolation, depression. Intergenerational housing advocated.
Matsumoto et al. (2021)	Cross-sectional study. Multisite; Japan. Ethnography; public housing complex.	Use/nonuse of community amenities	Patterns of use: park, school, library, community meeting room, and retail amenities	Supportive civic spaces and amenities promote socialization among the aged.
Sulander et al. (2016)	Cross-sectional survey. Multisite; Finland. Three-year study, subjects aged 75 and older in central Helsinki: 456 men, 939 women	Fourteen outdoor behavior settings: city parks, cemetery, and botanical garden	Frequency of visitation behavior	Individuals who visit urban green spaces most frequently, within 230 m from home had lowest mortality.
(1b) Aging in place in one’s existing home				
Abramsson et al. (2016)	Randomized cross-sectional survey. Multisite; Sweden. Nationwide questionnaire of 3 million subjects aged 55 and older.	Housing types and attributes	Propensity to relocate to smaller dwelling as a function of age and lifestyle preferences	Lack of affordable options exist for those who wish to relocate from larger owner-occupied to rental housing.
Anarde (2019)	Policy review. Multisite; United States. Aging in place housing trends in rural contexts.	Owner-occupied, detached single-family dwelling	Factors that predict independent aging in place housing preferences	Freedom of choice to remain in one’s rural community preferred over relocating to suburban/urban setting.
Anderson et al. (2021)	Mixed method. Multisite; Sweden. Thirty households surveyed with residents 65 years and older. Interviews.	Floor plan analysis of apartment units built between 1990 and 2015	Preference/satisfaction	Dwelling size, number of rooms did not predict satisfaction. Autonomy/efficient adjacencies preferred.
Boge et al. (2017)	Comparative case study. Multisite; Norway. Twenty private dwellings.	Functionality of bathrooms	Personal hygiene behaviors	Personal hygiene spaces seldom adequately support individuals with dementia and/or the tendency to fall.
Demirkan (2007)	Research review. Multisite; Europe. Design, aging, and independent living.	Physical accessibility; universal design	Satisfaction	Mainstream housing in private sector exhibits little regard for the needs of the aged.
Maaoui (2018)	Research review. Multisite; United States. Four-year study of construction of accessory dwelling units (ADUs).	Intergenerational housing amenities	ADU local permitting processes	Revised ADU permitting processes warranted in support of intergenerational urban housing.
Pettersson et al. (2020)	Systematic research review. General. 2000–2016. Dwellings occupied by persons aged 65 and older.	Private dwelling housing	Predictors of residents in need of home-based RLTC services ability to live independently	Research needed on best practices to modify community-based housing types/aging in place.
Rowles (2018)	Policy review essay. Case studies. Multisite; United States. Frail aged.	Independent living housing types	Home-based assistive technologies	Lawton and Nahemow (1973) theoretical model remain useful in environment and aging housing research.
Van Steenwinkel et al. (2012)	Case study; qualitative. Multisite; Belgium. Interviews with elderly residents’ post-relocation.	Independent living housing types	Assessments of the meaning of home	Meanings of home predicated upon perceived housing appropriateness in later life, autonomy, and safety/security.
Wahl et al. (2009)	Research review. Multisite; longitudinal: 1997–2006.	Dwelling retrofitting	Frequency of accidents by type and severity	Empirical evidence remains inconclusive, although fall incidents remain most prevalent adverse outcome.
Wiles et al. (2011)	Case study. Multimethod; New Zealand. Interviews, two RLTC homes; 121 participants aged 56–92.	Physical attributes of home, neighborhood	Security, personal choice/control, and familiarity	Meaningful place-attachment is a critical aspect of successful aging in place.
Wiley et al. (2012)	Cross-sectional study. Multisite; United States. 25,000 multifamily transactions, 24 housing markets.	Dwelling location, urban context, age/condition	Market demand for senior-living apartments	Resident educational attainment and life expectancy predict supply/demand for aging in place housing.
(1c) Multigenerational dwelling strategies				
Albuquerque (2011)	Cross-sectional study. Multisite; Portugal: 1994–2001; 4,881 residents surveyed age 65 and older.	Type of dwelling, size, and location	Multigenerational households; relatives	Multigenerational households are a timeless phenomenon, increasing in popularity.
Bodkin & Saxena (2017)	Cross-sectional study. Multisite; United States. Interview with 10 homeowners over age 65.	Type of dwelling, size, and location	Multigenerational home sharing; nonrelatives	Third-party-arranged home sharing enables elder to remain at home with live-in match. Cost-effective.
Burgess & Muir (2020)	Case studies. Multisite; United Kingdom. Interviews with 29 aged homeowners.	Dwelling type, size, location, and condition	Factors influencing multigenerational living	The motivations for multigenerational living are diverse, including worsening housing affordability.
Easthope et al. (2015)	Cross-sectional study. Multisite; Australia. Survey/interview, three-generation households; 392 respondents.	Dwelling attributes	Personal control of shared household space	Social hierarchies exist surrounding perceived versus actual control in multigenerational residences.
Gale & Park (2010)	Case studies. Multisite; United States. Interview/survey, 10 households, respondents 65 years and older, offspring age 30 and older.	Dwelling attributes	Privacy as function of age-stratified territorial zones	Kitchens most preferred for socialization; bedrooms are the most private zone in multigenerational dwellings.
Gerards et al. (2020)	Cross-sectional study. Multisite; Belgium. Interviews with 396 respondents aged 65 and older	Dwelling retrofits/conversions	Success rate of single-family dwelling adaptation	Respondents generally view multigenerational housing as viable option to counter housing unaffordability.
Judd (2016)	Cross-sectional study. Multisite; Australia. Interview/survey, 392 elderly residents in multigenerational households.	Dwelling attributes	Satisfaction with housing adaptations	Dwelling design attributes are major determinant with regard to satisfaction with multigenerational living.
Liu (2017)	Case studies. Multisite; Australia. Interviews (advocacy groups, planners, and developers), Chinese-born migrants in Melbourne.	Dwelling design attributes	Ability of dwelling to concurrently support two to three generations	Societal attitudes and evolving family structures influence the decision to live multigenerationally.
Souralová (2020)	Case studies. Multisite; Czech Republic. Interviews with three generations in shared households.	Dwelling attributes	Factors associated with ownership/rental status	The homeowner often determines social hierarchies and territorial control of shared space.
Suleman & Bhatia (2021)	Research review/policy. Multisite; Japan, Canada, the Netherlands.	Dwelling attributes	Predictors of intergenerational housing satisfaction	Intergenerational housing supports reduced loneliness and isolation among the aged.
**(2) Residentialism**				
(2a) Design considerations and case studies				
Bergland & Kirkevold (2006)	Nonrandomized comparative study. Multisite; Norway. two RLTC homes; 26 respondents aged 65 and older.	Indoor and adjacent exterior spaces	Resident satisfaction, well-being, patterns of use	Supportive indoor and adjacent outdoor spaces promote well-being in the RLTC setting.
Burton & Sheehan (2010)	Cross-sectional study. Multisite; United Kingdom. 20 RLTC homes; 81 elderly respondents.	Identification of key design features	Well-being, satisfaction.	Residents unwilling to be critical of their own RLTC home. Easier to assess nondesign issues.
Calkins (2009)	Research review. General. Multisite; international. RLTC/dementia care; 2000–2009.	Special Care Units (SCUs) for dementia care	Resident, family/staff satisfaction; nosocomial infection rate	Positive effect of private bedrooms on SCU residents’ well-being, and higher satisfaction among staff/families.
Carr et al. (2013)	Policy review. Multisite; Canada. Universal design in RLTC homes.	Physical and sensory accessibility	Universal design, well-being, satisfaction	Universal design affordances are a key facet of successful aging.
Chaudhury et al. (2013)	Research review. Multisite; general. 22 articles.	Kitchen/dining spaces; design	Resident satisfaction	Dining environments foster cognitive orientation, safety/security, sensory stimulation, socialization.
Chaudhury et al. (2016)	Case study. Single site; Canada. RLTC home dining spaces. Observation/survey, 10 residents, 17 staff.	Facility renovation/retrofit	Satisfaction; patterns of use, caregiving	Occupants cited greater personal control, socialization options, more effective caregiving, post-renovation.
Gromark et al. (2020)	Case studies, policy. General. Multisite; international.	Architectural design trends	Impact of design attributes on health outcomes	Home-based RLTC will increase in future as alternative to institutional care settings.
Nagahama et al. (2016)	Cross-sectional study. Multisite; Japan. Elderly housing; 490 dwellings.	Floor plan configurations	Preferred unit layouts	Unit layout types assessed. Shared amenities identified in socialization and dining/kitchen areas.
Nasrallah et al. (2021)	Research review. Multisite; general. 44 articles/books.	RLTC home design	Loneliness, isolation, well-being	The physical environment influences self-perceptions of resident loneliness; multiple theories discussed.
Neylon et al. (2019)	Case studies/research review. Multisite; international; 57 articles reviewed; 21 renovation projects.	RLTC home renovation	Preferred renovation amenities	Lighting, furnishings, color/contrasted surfaces, wayfinding cues, noise control, flooring of high priority.
Potter et al. (2018)	Cross-sectional study. Multisite; United Kingdom. 510 residents.	Exposure to outdoor space	Depression symptoms	Physical environment did not predict depression symptoms, with exception of outdoor spaces where policies preclude their use by residents.
Rijnaard et al. (2016)	Research review. Multisite; mixed method, international, RLTC homes; 17 articles.	Functional amenities and design	Satisfaction, well-being	Sense of home/place influenced by degree of personal control, autonomy, privacy, socialization, and community.
Tsuchiya-Ito et al. (2019)	Cross-sectional study. Multisite; Japan. Survey: home-care services, 1,928 respondents aged 65 and older.	Condition of dwelling; physical accessibility	Well-being; functional support	Substandard dwellings associated with lower well-being, ADL performance, more intensive home-care support.
Wahlroos et al. (2021)	Nonrandomized comparative study. Multisite; Finland. 20 RLTC residences.	Facility attributes	Psychometric assessment based on S-SCEAM-Fin. scores	The assessment tool was effective in comparing facility attributes of RLTC homes.
Wei et al. (2021)	Cross-sectional study: Multisite; China, RLTC homes.	Floor plan types, viewing distance, angle, visual field, and door orientation	Optimal bedroom configuration	Measurement tool was effective in analyzing bedroom spatial properties.
(2b) Personal space and cultural factors				
Cao & Dewancker (2020)	Cross-sectional study. Multisite; Japan. Observational, plan analysis, RLTC homes, nine prefectures.	Facility design and layout	Proxemic adjacency analysis	Preferred architectural plan typologies and optimal adjacencies identified.
Cao et al. (2021)	Cross-sectional study. Multisite; Japan. 168 RLTC facilities. Observational, no residents surveyed.	Floor plan layout	Preferred corridor configurations	Syntax theory/method provides designers with insight into spatial preferences among the aged.
Cater et al. (2021)	Cross-sectional study. Multisite; United States. Observational; 632 residents in RLTC facilities, assisted living, adult foster homes.	Floor plan analysis	Social cohesiveness, autonomy, control, engagement with physical environment	Organizational policies combined with spatial amenities predict perceptions of home.
van Hoof et al. (2015)	Case study. Single site; the Netherlands. Interviews, 12 RLTC home respondents; photo-diaries.	Photographs of RLTC facility	Personalization behavior	Residents’ self-documentation effective; architects can learn lessons from this participatory design method.
van Hoof et al. (2016)	Cross-sectional study. Multisite; the Netherlands. Interviews/observation, five RLTC homes, 27 respondents.	Spatial configuration	Personalization behavior	All bedrooms were embellished with personal artifacts; larger bedrooms preferred as they allow for furnishings.
Verderber & Song (2005)	Research review. General; Japan. Environment and aging, 1985–2002; 109 publications.	Activities of daily living (ADLs), relocation, lighting, and universal design	Therapeutic design factors	East-West research on architectural design trends, aesthetics, functionality, tectonics is advocated.
Verderber et al. (2020)	Case studies/policy review. Multisite; RLTC in Canada’s Far North; site selection, salutogenic design.	Elderhousing	Facility design prototypes	Culturally sensitive RLTC design advocated; mix of prefab off-site with on-site construction.
Yamaguchi (2020)	Case study. Experiment; Multisite; Japan. Two RLTC homes; videographic analysis.	Bed-space proxemics	Space requirements	Recommended standards for bed-space core functions: hygiene, bed placement, bed-to-wheelchair transfer.
**(3) Nature and landscape**				
(3a) Biophilia/therapeutic gardens				
Bengtsson & Carlsson (2006)	Nonrandomized comparative study. Multisite; Sweden. Three RLTC homes; interviews.	Outdoor spaces	Human comfort, satisfaction	Outdoor space preference a function of perceived fear of the outdoor realm and perceived affordances.
Bengtsson et al. (2015)	Cross-sectional study. Multisite; Sweden. Survey/interview, 26 aged residents.	Unbuilt versus built outdoor space	Semantic environmental description (SED); comparative assessment	Preferred outdoor settings are park-like, large, with varied vegetation.
Chi et al. (2020)	Research review. General. International. 137 articles/books.	Person–nature transaction; healthcare settings	Natural environment (NE) construct; occupant well-being	Five NE themes identified with implications for occupants’ physical and mental health, well-being.
Cooper Marcus & Sachs (2014)	Research review/book. Multisite; general; international. Best practices; theory/design.	Therapeutic and restorative landscapes	Stress reduction, psycho-emotive equilibrium, recovery from illness injury	Participatory strategies presented on garden design, maintenance, and optimal patterns of use.
Cutler & Kane (2005)	Cross-sectional study. Quality of life data, 1,988 RLTC residents; staff assessments.	Outdoor spaces	Access to the outdoors	Quality of outdoor space alone is insufficient predicator of usage. Physical ability is a more salient predictor.
Dahlkvist et al. (2016)	Cross-sectional study; multisite; 290 aged participants, 72 RLTC homes.	Outdoor garden with water element; physical access	Patterns of use; satisfaction	Navigable gardens with ample vegetation are most fascinating and preferred.
Edwards et al. (2013)	Case study. Single site; Australia. Interview/survey; 10 dementia SCU residents/staff.	Therapeutic garden	Satisfaction; health status	Reduced agitation/depression among study participants exposed to garden-atrium; increase in quality of life.
Eren et al. (2020)	Cross-sectional study. Turkey. 130 RLTC residents, seven RLTC settings.	Therapeutic gardens	Psychosocial well-being	Increase in satisfaction level with the outdoor garden results in greater psychosocial well-being.
Finlay et al. (2015)	Cross-sectional study. Multisite; Canada. Interviews, 141 RLTC home respondents aged 65–86.	Green (land) and blue (water) spaces; urban environment	Person–nature engagement	Accessible outdoor gardens are preferred as a direct means to improve residents’ quality of life.
Freeman et al. (2012)	Case study. Canada; 4-month pilot program in co-designed hydroponic gardening in RLTC facility.	Therapeutic garden	Satisfaction; physical activity	Active and passive engagement in gardening benefits residents across a diverse range of ability levels.
Gonzalez & Kirkevold (2016)	Cross-sectional study. Multisite; Norway. Online survey of 121 administrators.	Outdoor sensory gardens	Satisfaction, use, amenities	Preferred features: stable walkways, visible landmarks, accessibility, visibility from indoors, and prosthetic supports.
Hsieh et al. (2021)	Experiment. Multisite; Taiwan. Cognitively impaired; virtual reality (VR), two RLTC homes.	Therapeutic gardens	Length of exposure; heart rate	Length of exposure to a therapeutic garden is positively related to reduce stress level.
Ottosson & Grahn (2006)	Experiment/case study. Single site; multimethod; Sweden. 15 respondents aged 65 and older; seven in control group.	Outdoor garden	Blood pressure and heart rate	Physiologic measurement of concentration rates most increases following exposure to outdoor garden.
Peters & Verderber (2022)	Research review. Multisite; international. 109 articles; design for aging.	RLTC physical settings Biophilic design features	Satisfaction, well-being, staff performance	Person–nature engagement preferred by residents, families, and caregivers. Biophilia precepts presented.
Peters & Parekh (2022)	Research essay. Multisite; INTERNATIONAL	RLTC physical settings Biophilic design features	Satisfaction, well-being	Biophilia-based design affords opportunities to promote spatial orientation, improve residents’ mood and outlook.
Raske (2010)	Case study. Single site; United States. 96-bed RLTC home; 43 interviews: residents, staff, families.	Exterior garden; rural setting	Exposure; use/daily activities	Outdoor gardens support the Eden Alternative Model: empowerment, normalization, companionship, and flexibility.
Rodiek et al. (2016)	Nonrandomized comparative study. Multisite: United States. 152 outdoor spaces sampled; psychometric instrument tested.	Outdoor nature/landscape	Seniors Outdoor Survey: usage, preferences	Psychometric instrument with 60-item scale developed across five domains for outdoor garden assessment.
Van den Berg et al. (2021)	Quasi-experiment. Single site; the Netherlands; hospital geriatric ward.	Plants, related “greening” interventions	Sensory stimulation level; positive distraction source	Reduced length of stay, heightened physician discharge assessment associated with nature engagement.
Vecellio et al. (2021)	Research review. Multisite; general, international. 500 articles, RLTC homes	Exterior gardens and related spaces	Microclimate/nature exposure: perceived versus actual health outcomes	Seniors not adroit at sensing overexposure to outdoor hot/cold extremes due to reduced thermal sensitivity.
Xie & Yuan (2021)	Case study/experiment. Single site; China. Interview/observation, 95 RLTC home respondents aged 65 and older; 28 staff.	Exterior gardens and related spaces	Systolic measurement; health status	Outdoor activity elder-friendly environments should include spaces that stimulate physical movement.
Yari et al. (2021)	Case study. Quasi-experimental. Single site; United States. Assisted living facility (ALF), 31 resident respondents.	Exterior courtyard	Satisfaction, patterns of use	Renovated courtyard fostered multisensory stimulation, more active use by residents.
(3b) Nature engagement and dementia				
Calkins (2007)	Cross-sectional study. Multisite; United States. Memory care unit; 17 respondents.	Exterior spaces: nature, landscape	Seasonal exposure; sleep pattern; agitation	Increased time spent outdoors resulted in modest improvement in sleep patterns.
Chapman et al. (2007)	Cross-sectional study. Multisite; United States. 28 staff, survey; 20 memory care units.	Outdoor wandering garden, associated space	Staff assessment; use of exterior space	Advocates staff making heightened therapeutic use of exterior spaces for dementia care.
Cooper Marcus (2007)	Research method. General. Assessment checklist for design of memory care gardens.	Therapeutic gardens, associated outdoor spaces	Wayfinding; spatial orientation, satisfaction	Alzheimer’s Garden Audit Tool is presented for use in RLTC facility planning and design.
Cohen-Mansfield (2007)	Cross-sectional study. Multisite; United States. Survey; dementia care, 320 RLTC homes.	Outdoor wandering parks	Utilization, satisfaction.	Preference, usage dependent on affordances including seating, signage, lighting, accessible paths, and gazebos.
de Boer et al. (2017)	Nonrandomized comparative study. Multisite; the Netherlands. Survey, observation; 115 RLTC homes.	Green Care Farms.	Satisfaction; patterns of use	Green Care farm residents frequently engage nature, socialization, less so in inward-focused outdoor settings.
Detweiler et al. (2008)	Case study/policy. Single site; United States. Dementia care, observation, 34 residents, pre-post condition.	Renovated wandering garden	Usage; satisfaction; policy	Wandering garden usage resulted in significantly less agitation among residents, more positive mood.
Detweiler et al. (2009)	Case study/policy. Single site; United States. Dementia care; 28 respondents: number/severity of falls.	Outdoor wandering garden	Medications; incidence of falls; mortality rate	A 30% reduction recorded in falls; reduced high-dose antipsychotics; no change in related medications.
Grant & Wineman (2007)	Cross-sectional study. Multisite; United States. Dementia care; five RLTC homes.	Outdoor wandering gardens	Usage; satisfaction; policy	Organizational policy, staff attitudes, visual access, physical access, and garden design influence use.
Hernandez (2007)	Case studies/policy. Multisite; United States. Dementia SCUs; interview, behavioral mapping,	Therapeutic gardens	Usage, satisfaction, policy	Therapeutic gardens warrant incorporation as a standard element in SCUs for residents with dementia.
Kwack et al. (2005)	Research review. Multisite; general. Europe; dementia residents.	Wandering gardens	Satisfaction, exposure; usage	Provide safe, secure outdoor gardens with multisensory stimulation for the cognitively impaired.
Motealleh et al. (2019)	Research review/policy. General. 15 studies. 1,179 participants (residents, staff, family). Residents aged 71–89.	Outdoor wandering landscape	Agitation, apathy, nature engagement	Further research needed on impacts of outdoor natural landscapes on residents’ behaviors and health status.
Rodiek & Schwarz (2008)	Applied theory. General. Guidelines and policy.	Therapeutic gardens, wandering gardens	Satisfaction, well-being; training	A book on theoretical perspectives and applied case studies for designers as well as direct caregivers.
Scott et al. (2022)	Systematic review. Horticulture therapy, RLTC settings. Community-based aging in place study participants.	Outdoor gardens	Social cohesiveness; engagement with physical environment	Horticultural therapy affords multiple positive benefits for persons across a broad range of ability levels.
Whear et al. (2014)	Research review/policy. General. Dementia care; 17 studies.	Therapeutic gardens, associated outdoor spaces	Exposure; agitation; satisfaction	Health status (agitation) is predicted by immersion level/exposure to gardens, associated policies.
**(4) SCUs**				
(4a) Immediate living spaces				
Barrett et al. (2019)	Theory/policy. General. Multisite. Review of best practices.	SCUs	Well-being, satisfaction, health status	The built environment aids in fostering cognitive clarity and sequencing.
Campo & Chaudhury (2012)	Cross-sectional study. Multisite; Canada. Two RLTC homes; 43 dementia care study participants.	Home-like features, visual sightlines	Well-being, preference	Well-being is predicted by seating in public areas, visual sightlines, lighting, respite, identifiable spatial realms.
Charras et al. (2016)	Cross-sectional study/policy. Multisite; France. Survey, observation. Three SCUs, dementia care.	Facility floor plans, design features	Satisfaction, well-being	Four principles of eldercare human rights should guide facility design: respect, freedom, dignity, and equality.
Chaudhury & Cooke (2014)	Policy review/essay. General. Book chapter. Quality of life in RLTC homes.	Unit size, image, dining space, resident room, bathing/toileting, and outdoor space	Spatial disorientation, anxiety, agitation, and social withdrawal	Greater design attention is warranted to eliminate environmental stressors for the cognitively impaired.
Chaudhury et al. (2018)	Research review. General. Dementia care; 94 empirical studies, nine essays.	Unit size/layout, home-like aesthetic, environmental cues	Personal control, sensory stimulation	Further user-centric research and policies warranted to improve environments for the cognitively impaired.
Chaudhury et al. (2013)	Research review; multisite; general. 22 studies; mixed methods.	Dining areas; associated spaces	Assessment of home-like affordances	Dining spaces should foster spatial orientation, safety/security, sensory stimulation, personal control.
Garre-Olmo et al. (2012)	Cross-sectional study. Multisite; Spain. 160 RLTC homes.	Temperature, noise, lighting levels	Pain management, neuropsychiatric disorders	High temperature, low lighting levels in bedrooms associated with decreased well-being.
Marquardt (2011)	Research review. General; international. 169 articles.	Architectural unit types, design attributes	Satisfaction, cognitive functioning	Redundant cued wayfinding amenities promote well-being in dementia care units.
Marquardt et al. (2014)	Research review/policy. Multisite; Germany. Dementia care SCUs; 169 studies.	Lighting, noise, room temperature, color/form, imagery, ambience, and environmental cues	Cognitive functioning, well-being	Strong relationship exists between built environment, physical functioning, socialization, and cognitive orientation.
Marquardt and Schmieg (2009)	Cross-sectional study. Multisite, Germany. 30 RLTC homes, dementia care.	Interior circulation, exterior courtyards	Wayfinding, cognitive orientation	Residents with advanced dementia are most dependent on focused wayfinding amenities.
Molony (2010)	Summary review, meta-ethnography; residential transition.	Key design attributes	Meaning of home	Articulation of theory framework to guide residents’ transition from home to institutional setting.
Olson et al. (2021)	Research review/case studies. Multisite; international; dementia care, COVID-19	Dementia SCU facilities	Wayfinding, elopement, falls/injury, pharmacological intervention, and mortality.	Overcrowded, poorly configured dementia SCUs facilitate the spread of COVID-19.
Slaughter et al. (2007)	Cross-sectional study. Multisite; Canada. Comparative; SCU versus traditional RLTC unit.	Maintenance, safety, orientation cues, lighting, noise, and image	Therapeutic Environment Screening Scale (TESS-21); SCU Quality Scale (SCUQS); well-being	Few significant differences identified between SCU and TESS scores
Tartarini et al. (2017)	Experiment/longitudinal study. Single site; Australia. Indoor temperature, dementia care: 21 residents studied across 10 months.	Indoor air temperature: 16.2 °C–33.6 °C	Agitation, health status	Agitated behaviors were statistically correlated with temperature levels experienced beyond 20 °C–26 °C.
Verbeek et al. (2010)	Cross-sectional/controlled experiment. Multisite; the Netherlands; 124 participants: two groups.	28 small-scale facilities; 21 psychogeriatric care units	Neuropsychiatric symptoms, agitation	Study was unable to demonstrate affirmative benefits of small-scale SCUs for dementia care.
van Hoof et al. (2009)	Policy review/essay. Multisite; Europe. Aging-in-place dementia care; financing options.	Independent living dwelling typology	Well-being, preference	Countries providing a high level of services for the aged with dementia share similar aging in place policies.
van Hoof et al. (2010a)	Case study. Multimethod; interviews, literature review.	Indoor air quality	Thermal comfort/discomfort	Thermal comfort is fundamental in the care of persons with dementia, who have specific needs.
van Hoof et al. (2010b)	Literature review; general, RLTC homes.	Indoor environment	Impact of design attributes on dementia residents	Design attributes of basic value, functional value, and economic value are reported.
Wong et al. (2014)	Case study. Single site; Australia. Indoor air temperature: 21 RLTC home residents with dementia.	Indoor temperature	Agitation; length of exposure	Preferred indoor air temperature levels reduce agitation and stress among residents with dementia.
Yokoyama et al. (2009)	Cross-sectional study. Multisite; Japan. Floor plan analysis, behavioral observation.	Spatial properties	Engagement in ADLs	Meals/cooking, relaxation/sleeping, personal hygiene, praying, and therapy activities predict well-being.
(4b) Design interventions				
Burke & Veliz Reyes (2021)	Qualitative study; comparative case study: United Kingdom. Two RLTC homes, each with 10–50 dementia care residents.	Residential amenities	Functional performance, well-being	Grounded theory provides useful basis for assessing person-environment fit in dementia care settings.
Cadigan et al. (2012)	Nonrandomized comparative study. Multisite survey; United States. 323 participants, 22 SCUs versus traditional RLTC units.	SCUs	Tube feeding, hospitalizations, satisfaction	Higher quality of end-of-life care is provided in SCUs for persons with cognitive impairment.
Calkins (2018)	Research review. General. Book chapter	Spatial configuration; design	Self-actualization, autonomy, safety/security, connectedness	Settings that minimize stress, support autonomy and functional independence, promote well-being.
Caspi (2014)	Cross-sectional study. Multisite; United States. SCUs and assisted living compared; dementia care, 13 interviews.	Floor plan configuration; associated features	Wayfinding behavior, well-being	Small-scale memory care environments with 7–10 residents per household are advocated.
Connell et al. (2007)	Case study. Single site; United States. Comparative: 20 dementia residents aged 65 and above.	Indoor/outdoor space, design	Activity level, sleep patterns, and agitation	Outdoor activity group: improved sleep pattern/less agitation. Indoor group: improved sleep pattern only.
Davis et al. (2009)	Case studies/policy. Australia. General; comparative analysis.	Floor plan analysis, design	ADLs	Relocation from traditional unit to memory care SCU has positive effect on well-being and quality of life.
de Rooij et al. (2012)	Cross-sectional experiment. Multisite. Belgium, the Netherlands. Comparative, 179 residents with dementia.	Physical setting attributes small-scale facilities versus traditional RLTC’s	Satisfaction, well-being	Small-scale settings foster the most diverse benefits for resident well-being and quality of life.
Eijkelenboom et al. (2017)	Cross-sectional study. The Netherlands, interior design, RLTC homes.	Interior design attributes	Satisfaction, well-being, staff performance	Checklist for practitioners is presented to enhance residents’ sense of home in RLTC settings.
Feng et al. (2018)	Case study. Single site; the Netherlands. Montessori-guided dementia care regimen; interview, observation.	Architectural setting; furnishings	Level of stimulation, agitation; engagement	Montessori-based amenities facilitated heightened satisfaction among study participants.
Ferdous (2021)	Case study/policy. Single site; United States. Eight memory care interventions, COVID-19.	Key indoor and outdoor design features	Loneliness, depression, mortality	Small-scale facilities, outdoor space, adaptability, social distancing, and air quality critical in COVID-19 treatment.
Fisher et al. (2018)	Case study, dementia care, United Kingdom. 10 residents; five staff. Semi-structured interview.	RLTC dementia care setting	Staff performance; resident well-being	Staff and resident built environment needs must be balanced to endure highest quality care.
Fleming & Purandare (2010)	Research review. Multisite; General. 57 articles 1970–2008.	Key design features	Safety/security, accessibility, personalization, sensory stimulation	Safety, smallness, privacy, visual access, sensory stimulation, wayfinding cues, nature high priorities.
Fleming & Bennett (2015)	Cross-sectional methodological study; diagnostic tool, general.	Memory care unit design	Resident well-being; health status	Environmental Audit Tool metric is presented to assess the performance of RLTC care settings.
Kok et al. (2016)	Nonrandomized comparative study. Multisite; the Netherlands. SCUs, 67 relocated residents, dementia care.	SCU unit configuration; architectural features	Cognitive functioning; well-being	No significant differences in cognitive functioning identified. Small-scale fosters highest well-being.
Lee et al. (2016)	Case study/policy. Multisite; Canada. Focus group, interview.	Memory care SCU physical setting	Physical comfort, well-being	Small-scale, therapeutic physical environments positively influence dementia residents’ quality of life.
Milke et al. (2009)	Cross-sectional study. Multisite; United States, Canada. Comparative, five RLTC homes; 184 participants; behavioral mapping.	Common areas, staff workspace, outdoor space, walking paths, upkeep	Patterns of use; cognitive orientation	Residents’ well-being is differentially impacted based on type/scale/residential amenity of care setting.
Morgan-Brown et al. (2013)	Cross-sectional study. Multisite; Ireland. Two RLTC homes, floor plan analysis.	Traditional versus open plan communal spaces	Usage, satisfaction, policy	Open plan communal spaces resulted in heightened social engagement. Policy implications discussed.
Orfield (2015)	Policy review. Multisite; international. Overview of recent trends in dementia care settings.	Physical setting attributes Key design features	Cognitive functioning; well-being	Memory care unit design supports residents’ capacity to translate environmental cues to interpretable patterns.
Pollock & Fruggle (2013)	Cross-sectional study, general, RLTC homes.	Spatial legibility and functional support	Patterns of use; cognitive orientation	A set of design provisions are presented for use by designers and direct caregivers.
Quirke (2018)	Theory essay/policy. General. RLTC settings.	Unit plan configurations	Satisfaction; performance	Adaptation of postoccupancy tools for use in dementia SCU settings.
Quirke et al. (2021)	Methodology study, general. Comparative assessment of design process tools for RLTC settings.	Memory care unit design	Efficacy of research tools	Adaptation of postoccupancy tools for use in dementia SCU settings.
Verbeek et al. (2009)	Research review. Multisite; international. 75 studies/essays on group living; dementia care.	Kitchens, dining areas	ADLs, agitation	Nutritional health spaces key to home-like atmosphere. Unit size not correlated with less agitation.
**(5) Voluntary/involuntary relocation**				
Abrahamson (2016)	Case study/policy. United States. Interviews, 20 family members.	Institutional relocation	Transfer process; family perspectives	Family members highly value resident’s physical setting comfort/physical amenities, and quality of life.
Aminzadeh et al. (2010)	Case study. Multimethod; Canada; interviews, 16 residents with dementia, prerelocation.	Long-time dwelling; institutional relocation	Cognitive disorientation; environmental stress	Strategies presented to facilitate transition from home to institution; pre- and post-relocation.
Capezuti et al. (2006)	Cross-sectional study/policy. Multisite; United States. Longitudinal, 23 RLTC homes: 120 residents; 56 family members.	Institutional relocation	Fall incidents; well-being	Relocation need not result in adverse physical/mental outcomes. High rate of falls occurred posttransfer.
Castle (2005)	Cross-sectional study. Multisite: United States; 6 year study; 301 RLTC home residents.	Institutional relocation	Health status; satisfaction	Relocation impacts identified: cognitive performance, depression, social engagement nonsignificant.
Cheek et al. (2006)	Policy review/essay. General; Australia. National; retirement villages, interviews, survey.	Institutional relocation	Well-being	Policy diversity advocated between retirement villages, with specific attention to individual facility affordances.
Cioffi (2007)	Case study. Single site; Australia. Interview, 21-bed SCU in 300-bed RLTC facility; seven relatives, 12 staff.	Memory care unit	Family/staff: environmental adaptation	The built environment influences residents’ functioning, sense of freedom, agitation, sleeping patterns, and weight.
de Boer et al. (2021)	Policy review/essay. General. International. Transition from home to RLTC facility, relocation from-to hospital.	Institutional relocation	Adverse impacts: three phases	RELOCARE findings: expand facility options premove, maximize education, and environmental and social supports.
Engberg & Castle (2008)	Nonrandomized comparative study. Multisite; United States: 12 RLTC homes; 439 residents.	Institutional relocation	Health status; well-being	Hurricane Katrina adverse relocation impacts identified: ulcer rate, mortality increased.
Falk et al. (2011)	Cross-sectional/experiment. Multisite; Sweden.155 participants; 74 relocated; 81 in control group, two RLTC homes.	Institutional relocation	Health status; well-being	Relocated residents’ health deteriorated significantly versus nonmovers. Relocation unpredictable, stressful.
Hagen et al. (2005)	Experiment. Multisite; Canada: Comparative; three RLTC homes, 289 residents.	Institutional relocation	Antipsychotic medications; usage	Among the nonrelocated, 31.3% residents received antipsychotics, significantly less than relocated cohort.
Holder & Jolley (2012)	Research review/policy. Multisite; general. RLTC home closure/involuntary relocation. 108 articles reviewed.	Institutional relocation	Health status; well-being	Ill-planned facility closure and involuntary resident relocation linked to lower health status, mortality.
Innes et al. (2011)	Cross-sectional study. Multisite; Northern Ireland, Scotland. Focus groups: 29 with dementia; 11 family carers.	Memory care unit	Family and resident satisfaction	Physical design is a key dimension in daily living: wayfinding cues, amenities, and outdoor space use.
Jolley et al. (2011)	Policy review. Multisite; General. European Court of Human Rights (ECHR) ruling; eight articles reviewed.	Institutional relocation	Health status	The built environment represents key aspect of the total scope of prerequisite services and supports.
Kelsey et al. (2009)	Cross-sectional study. Multisite; United States; interviews, 37 ALF administrators.	Assisted living facilities; relocation	Health status	Administrators need to better inform families of transfer policies to CCRCs upon resident’s first admission.
LaMantia et al. (2010)	Research review/policy. International. Five studies.	RLTC-to-hospital relocation	Medication dosage/duration: pre-/posttransfer	Research needed to further define the most impacted residents relative to facility context, outcome measures.
Laughlin et al. (2021)	Experiment. Multisite; United States. Relocated residents (*n* = 83) and nonrelocated (*n* = 90)	Institutional relocation	Health status; mortality	Relocation-related stress was sole significant predictor of higher mortality rate among the relocated cohort.
McFadden & Lunsman. (2010)	Cross-sectional study. Multisite; United States. Interview, observation; 22 dementia care subjects, from traditional to two smaller units.	Institutional relocation	Social/anti-social behavior	Few significant differences among residents relocated from traditional to small-scale RLTC residence.
Williams et al. (2007)	Case study/policy. Multisite, United Kingdom. Eight facility closures. Administrators, pre-/postinterview.	Institutional closure	Relocation; health outcomes	Public agencies need to exert greater influence to mitigate adverse outcomes for residents, staff, and families.
Wu & Rong (2020)	Case study. Multisite; Taiwan. Interviews, 16 post-relocation elderly persons in RLTC home for 12 months.	Institutional relocation	Well-being, adaptation, and satisfaction	Relocation to RLTC facility is a dynamic process in the first year postmove, requiring extensive support.
Yamada et al. (2014)	Cross-sectional study. Multisite; Japan. Interviews, observation; two apartment buildings for seniors.	Relocation to noninstitutional dwelling	Well-being, satisfaction	Reluctant cohort: negative impacts; volunteers did not. Unit design, direct access to outdoors predict outcome.
Yamamoto (2008)	Cross-sectional study. Multisite; Japan. Eight cities; survey questionnaire: 1,970 respondents.	Institutional relocation	Satisfaction	Well-being influenced by income level, physical mobility, facility supports, access to local community resources.
**(6) Infection control/COVID-19/environmental stress**				
(6a) Ambient conditions, safety, and infection control				
Barrick et al. (2021)	Nonrandomized comparative study. Multisite; United States. Dining areas, geriatric psychology dementia SCU; 60 participants.	Light type/quality	Agitation incidents, intensity	Ambient bright lighting ineffective in reducing agitation in dementia SCU; may exacerbate adverse outcomes.
Bentayeb et al. (2015)	Cross-sectional study. Multination; Europe. Survey, medical exam; 600 respondents 65 and older, 50 RLTC homes.	Indoor air quality	Respiratory health status	Results verified that low level, poor indoor air quality affects respiratory health frailty, increasing with age.
De Lepeleire et al. (2007)	Cross-sectional study. Belgium; Observation; eight RLTC homes; 16 behavior settings.	Lighting type/quality	Visual acuity; visibility	Light levels in RLTC homes insufficient to meet the visual needs of residents, posing hazardous daily risk.
Dowling et al. (2005)	Randomized control trial. Multisite; United States. 70 participants; two RLTC homes.	Lighting type/quality	Sleep-rest patterns	Residents exposed to ambient bright light conditions experienced more normative sleep-rest patterns.
Fetveit & Bjorvatn (2005)	Experiment/pilot study; Single site; Norway. 11 RLTC residents; two weeks. Observation, staff diaries.	Lighting type/quality	Sleep-rest patterns	Bright light exposure effective in reducing daytime sleep duration among dementia residents.
Friedman et al. (2012)	Cross-sectional study. Multisite; United States. 54 caregivers, two week study period.	Lighting type/quality	Sleep disturbance patterns	Phototherapy reduced sleep duration among cognitively impaired with insomnia, diagnosed depression.
Garcia et al. (2012)	Cross-sectional study. Multisite; United States. Focus groups, eight units: 45 family members; 59 staff.	Memory care unit	Family/staff views: socialization; well-being	Noise control identified as key environmental stressor influencing residents’ behavior and daily quality of life.
Giggins et al. (2019)	Experiment/pilot study. Single site; Ireland. 10 residents; activity monitor scores recorded.	Lighting type/quality	Sleep patterns; mood	Cycled lighting therapy proven an effective intervention among the institutional aged.
Hickman et al. (2007)	Cross-sectional study. Multisite; United States. 66 older adult participants; four ambient lighting conditions.	Lighting type/quality	Depression; mood	Ambient bright light therapy generally proven ineffective for reducing depression among dementia patients.
Jiang et al. (2021)	Nonrandomized comparative study. Multisite; China. Survey: 739 respondents aged 65 and older; 25 facilities.	Facility accessibility	Fall incidents; well-being	The most common hazards are inadequate, inappropriate handrails, unsafe flooring, and poor lighting.
Joosse (2011)	Cross-sectional study. Multisite: United States. Sound levels, eight RLTC homes; 424 observations recorded.	Residential setting noise	Sound intensity; agitation	Sound levels were generally high: 27% of talking-based noise on unit was not directed at residents.
Joosse (2012)	Cross-sectional study; Multisite; United States. 53 participants; four RLTC homes.	Residential setting noise	Stress; agitation	High-noise exposure level resulted in environmental stress among dementia residents.
Kim et al. (2021)	Cross-sectional study. Multisite; United States. 57 participants; two RLTC homes.	Lighting type/quality	Satisfaction	Normative experimental conditions recorded. Intensive design attention to illumination is recommended.
Kovach et al. (2017)	Experiment. Single site; United States. 160-bed RLTC home; room modification.	Ultraviolet lighting; anti-microbial surfaces	Incidence of infections; new equipment	Pulsed-xenon ultraviolet disinfection method proven superior to manual room cleaning in preventing infection.
Riemersma-van der Lek et al. (2008)	Nonrandomized study. Multisite; the Netherlands; 189 dementia residents, 12 RLTC homes; mean age 85.8.	Lighting type/quality	Cognitive dissonance, sleep, and daily activities	Bright light therapy exerted modest positive impact in mediating cognitive/noncognitive dementia symptoms.
Royer et al. (2012)	Experiment. Single site; United States. Double-blind placebo design: RLTC home, 15 “treated,” 13 placebo subjects.	Lighting type/quality	Sleep patterns; mood	Blue light therapy treatment resulted in significant cognitive improvement, compared to placebo red light.
Sloane et al. (2007)	Cross-sectional study. Multisite; United States. Four conditions, two RLTC homes: 40 residents.	Lighting type/quality	Sleep patterns; mood	Bright light therapy (mornings) has positive effect on sleep patterns; ambient light preferred versus fixtures.
Stone et al. (2015)	Cross-sectional study/policy. Multisite; United States. 10 RLTC homes; survey; 78 study participants.	Facility-based infection control	Efficacy of infection control protocol	Standardized infection control policies proven effective in improving the quality of the physical environment,
Tartarini et al. (2017)	Experiment. Single site; Australia. Longitudinal; 21 residents; 10-month study.	Indoor air quality	Cognitive dissonance; agitation	Temperature level beyond the comfort range of 20 °C –26 °C causes agitation; temperature variations should be limited.
Thomas et al. (2020)	Research review/pilot study. General; international. four studies; staff interviews	Noise/acoustics	Well-being; satisfaction	Preferred noise/acoustical properties of RLTC settings must take use preferences into greater consideration.
White et al. (2013)	Research review. General. 48 articles; randomized trials, spectral-timing sequencing.	Lighting type/intensity	Circadian rhythms; sleep disorders	Programmable 24-hr light/dark ambient condition cycles help to mitigate circadian rhythm disruption.
Yasuda & Miura (2021)	Experiment. Single site; Japan; Floor plan, observation; 12 resident rooms.	Spatial configuration/visual barriers	Sightline efficacy	Direct sightlines essential for providing high-quality care, balanced with resident privacy, personal autonomy.
(6b) COVID-19				
Anderson et al. (2020)	Policy review/essay. Multisite; General. COVID-19, RLTC home; adverse health impacts.	Residential setting design	Health status; mortality	Architectural resiliency, flexibility, and design excellence determines the well-being of residents and staff.
K. A. Brown et al. (2021)	Cross-sectional study. Multisite; Canada. 78,000 residents; 600 RLTC homes in Ontario.	Residential setting design	Overcrowding; mortality	Overcrowding is directly associated with the majority of COVID-19 outbreaks.
Lynch & Goring (2020)	Research review/policy. General; United States. CDC database on indoor air quality.	HVAC filtration systems	Airborne infection rates	Negatively pressurized rooms, dedicated exhaust portals, improved filtration, and closed doors are essential.
Olson & Albensi (2021)	Literature review. General. International. 69 articles. COVID-19, dementia care, lighting, noise, wayfinding.	Memory care unit design	Health status; mortality	Single bedrooms reduce agitation, aggression, improve sleep quality, reduce risk of falls, restraints, and medication.
Thompson et al. (2020)	Qualitative case studies: multisite: Australia, Europe, and United States. COVID-19 related deaths in RLTC homes.	Residential setting design	Health status; mortality	Discrepancies between public and private sectors identified include facility construction underfunding.
Z. Wang (2021)	Policy review. Multisite; China. COVID-19 controls; RLTC units. Interviews with six administrators.	HVAC filtration systems	Health status; mortality	Infection control must be high priority. Future designs, retrofits must be guided by the COVID-19 experience.
Zhu et al. (2022)	Cross-sectional study. Multisite; United States. 7,785 RLTC homes surveyed: 50.8% Medicare and/or Medicaid recipients.	Residential setting design	Health status; mortality	Private bedrooms, hygiene, and common living areas must be highest priority in mitigating infectious disease.
**(7) Sustainability/facility management**				
Calkins & Cassella (2007)	Research review/policy. Multisite; United States. Interviews/plan analyses: four RLTC home administrators; four architects.	Resident bedroom/bath-shower room	Three bedroom types compared; health outcomes	All-private bedrooms yield improved health outcomes versus shared bedrooms and are more cost-effective.
Ivanko et al. (2020)	Nonrandomized comparative study. Multisite; Norway. Three RLTC homes; patterns of use.	Hot water heating system	Hot water usage; residents’ hygiene/comfort	Hot water/heat usage in winter highest. Maximum hot water heat use occurs 9–11 a.m., minimum use 2–5 p.m.
Peterson et al. (2014)	Policy Review. General; United States. Book chapter. Adverse events.	Disaster preparedness	Health status; mortality	Recommended infrastructure protocols: pre-risk assessment; pre-tests/drills; tested evacuation plans.
Sun et al. (2020)	Case study/policy. Single site; United States. 12 RLTC home residents died; HVAC system failure.	Thermal resilience	Mortality rate; energy cost-savings	Hurricane Katrina: Attuned thermal resilience reduces heat exposure; passive natural ventilation cost-effective.
Teni et al. (2019)	Cross-sectional study. Multisite; Croatia. Survey/interview; three RLTC homes.	Pre-post renovation processes; energy consumption	nZEB-related facility management	nZEB policies result in improved thermal performance, and yield cost-savings.
Verderber & Peters (2019)	Case study: Book chapter. Multisite; United States, Canada. 18 LEED RLTC homes. Observation, archival analysis.	Ecological, biophilic, salutogenic design	Sustainability; quality of life	LEED certification alone does not guarantee broader, ecohumanist architectural design excellence.
**(8) Design trends/prognostications**				
(8a) Green house model (GHM)				
Afendulis et al. (2016)	Case studies. Multisite; United States. GHMs compared to 223 matched non-GHM RLTCs.	Key design features	Rehospitalization; health status	GHMs result in lower hospital readmission rates, fewer medications, ulcer rates among residents.
P. B. Brown et al. (2016)	Cross-sectional study. Multisite; United States. 13 GHM facilities compared with Eight conventional RLTC homes; 11 states.	Comparative assessment of key design features	Satisfaction; performance	Lower staff turnover, higher satisfaction levels among GHM staff. Few other significant differences identified.
Cohen et al. (2016)	Cross-sectional study: Multisite: United States. Interviews; 12 GHM facilities.	Preferred unit design strategies	Satisfaction; health status	Variations on the GHM are emerging, making it difficult to compare resident health, staff performance levels.
Cutler & Kane (2009)	Case study. Single site; United States. Four GHM facilities. Mapping, traces, survey, and interview.	Comparative assessment of facility design features	Postoccupancy evaluation; satisfaction	GHM residents tend to remain in own room for varied activities, including visitations, often with door closed.
Fishman et al. (2016)	Research review/policy. General. International. Expert panel review of GHM.	Residential unit spatial design features	Staff and resident satisfaction, performance	Expert consensus: private bedrooms, access to nature, privacy, and autonomy highly effective.
Kane et al. (2007)	Cross-sectional study. Multisite; United States. Four 10-person GHM residents, staff, observation, and interviews.	Comparative assessment of facility design features	Administrator ratings; staff, resident well-being	Quality of life, built environment ratings generally favor Green House facilities over traditional RLTC’s.
(8b) Future trends				
Craig (2017)	Case study/policy. Multisite; United Kingdom. Three RLTC homes; video facility documentation, interview.	RLTC residences	Well-being; health status	Essay on the importance of design innovation in eldercare built environments.
Engelen et al. (2022)	Research Design, Setting and Sample	Salient design features; indoor environmental quality	Quality of life; therapeutic supports	7 themes identified including person-nature transactions, wayfinding; limited empirical support for safety/security, setting adaptability.
Kerbler (2016)	Case studies. Multisite. Europe, 65, Japan, South Korea, Singapore. Interview; two cohorts, 57 participants over age 65.	RLTC residences and in-home-based care	Well-being; mortality	Rapid innovation is needed in built environments for eldercare, but society will be slow to respond.
Lundstedt et al. (2021)	Case studies/policy. Multisite; Sweden. Interview, observation, VR.	Virtual NEs	Well-being; VR skill development	VR technology is a creative adjunct in eldercare everyday settings, improving residents’ quality of life.
Nasrallah & Patti (2021)	Research review. General. International; 44 articles/books.	Key unit design features	Well-being; prognostications	Seven environment and aging theories comparatively analyzed, providing insights into person-place interface.
National Academies of Science, Engineering & Medicine (2022)	Research/policy review. General, interviews, focus groups, US congressional hearing.	Spaces that promote privacy and independence	Well-being; future design priorities	Chapter 6: “Nursing Home Environment and Resident Safety” stresses infection controls, improving quality of life.
Orfield (2013)	Case studies/policy essay. General. Nongovernmental organization best practices in RLTC built environments.	Physical setting/design, perceptual aspects of facility design	Satisfaction; well-being	Methodist Homes and Action Pact integrates physical, financial, cultural, and futurist operational elements.
Pirinen (2016)	Case studies/essay. Multisite: Finland. User participation.	Communal senior housing, assistive features	Architectural design best practices	Resident-initiated and noninitiated elderhousing policies are contrasted, with recommendations presented.
Regnier (2018)	Comparative Case study/policy. Multisite: the Netherlands, United States. Interviews, site visits, literature review.	Residential group living units	Well-being, mortality rates	Dutch apartments assessed as comparable to Green Houses. Guidelines presented on care of the old-old.
Schwarz (2012)	Policy review/essay. General. International best practices in eldercare.	Assistive physical design features	Health status; satisfaction; adaptability	Environmental gerontology is solution-driven, with overemphasis on applications, versus guiding theory.
van Hoof et al. (2014)	Experiment/case study. Multisite; the Netherlands. 22 mind mapping sessions/97 elderly participants.	Private rooms and adjacent living areas	Cognitive visualization; mapping	Dutch nursing home design can benefit from predesign visualization research based on systematic user input.
C. Wang & Kuo (2006)	Policy essay/theory. Taiwan. Interview; RLTC staff/administrators; Delphi method.	Key residential unit design features	Adaptability, sustainability, and resilience	Priorities for future: residential, universal design; private bedrooms; nature; socialization space; and decentralization.

## Results

### Community-Based Non-RTLC Settings

#### Immediate neighborhood and urban environment

The contributing role of the community context, including physical infrastructure and the potentialities of multigenerational housing, has received increasing evidence-based research attention. Studies address the role of the residence in relation to walkable and transit-accessible amenities, as well as the function of home-based independent living. User need assessment methods are increasingly being utilized to identify age-appropriate community context amenities for the aged who are unable to continue to live independently (Alley et al., 2007; Aung et al., 2021; Baldwin et al., 2013; Buffel et al., 2012). Older persons who live alone are particularly vulnerable to loneliness and isolation and are in need of a safe, walkable neighborhood (Forsyth et al., 2019). Matsumoto et al. (2021) studied a 17,000 resident social housing complex in Japan with one third of its residents aged 65 and older. Six frequent patterns of behavior were identified: residents who visiting the nearest local park, those who volunteered at the local school, residents who frequented the nearest public library, those who utilized meeting rooms on site, and residents who engaged in shopping and attending cultural events in the local neighborhood. With respect to CCRCs, the on-site presence of comparable amenities and the ability to directly access additional amenities in the neighborhood were found to be the source of satisfaction to residents (Campbell, 2015). In a multisite study in Finland, Sulander et al. (2016) found that older persons who live independently and most infrequently visit local urban green spaces, including parks, a botanical garden, and a local cemetery, experienced the greatest risk of mortality as a function of their isolation and physical inactivity.

#### Residing in one’s existing home

Continuing to reside in one’s longtime residence has implications far beyond the mere provision of housing per se (Wiles et al., 2011). In a study conducted in Sweden, Andersson et al. (2021) concluded that small apartments for independent living are most preferred as they allow for personalized adaptation—if they are internally well-planned from the outset. In a related study, accessory dwelling units proved a valuable addition to housing options for older persons (Maaoui, 2018). With respect to the relationship between market forces and housing older persons, the household downsizing trend in many countries, combined with emergent independent living preferences has been examined (Abramsson & Andersson, 2016; Demirkan, 2007; Wiley et al., 2012). It was concluded, in part, that acute shortages of age-appropriate housing options will intensify in the coming years unless anticipatory public policies are enacted in a timely manner to avoid a housing affordability crisis. Older persons who reside in rural communities continue to be particularly overlooked in this respect (Anarde, 2019). At the architectural scale of inquiry, numerous studies have examined adaptive measures to make residents’ long-standing homes more supportive of aging in place lifestyles (Pettersson et al., 2020; Rowles, 2018; Van Steenwinkel et al., 2012), and specifically, with respect to mitigating the occurrence of falls (Wahl, 2009). In closely related research, the intersection of privacy and personal hygiene has been researched in specific rooms within housing for older persons, that is, bathrooms (Boge et al., 2017).

#### Multigenerational dwelling strategies

The traditional pattern of grandparents living in multigenerational households has been the subject of considerable recent research, including in Portugal (Albuquerque, 2011), the United Kingdom (Burgess & Muir, 2020), Belgium (Gerards et al., 2020; Souralová & Žáková, 2020), and Australia (Liu, 2017). In a recent Canadian study, it was concluded the provision of community-based healthcare supports is essential for older persons in multigenerational residential settings (Suleman & Bhatia, 2021). Third-party-arranged home sharing has been field tested as a viable way for elders to remain in their homes by connecting them with suitable (often nonelderly) prescreened live-in matches (Bodkin & Saxena, 2017). Personal control, privacy, and autonomy are of high priority in older residents’ daily functioning in multigenerational living settings (Easthope et al., 2015), particularly in shared common spaces such as kitchen-dining zones (Gale & Park, 2010; Judd, 2016).

***Personal control, privacy, and autonomy are of high priority in older residents’ daily functioning in multigenerational living settings (Easthope et al., 2015), particularly in shared common spaces such as kitchen-dining zones (Gale & Park, 2010; Judd, 2016)***.

### Residentialism

#### Design considerations and case studies

The term *residentialism* has been defined as the late 20th and early 21st-century international movement to inculcate a home-like architectural environment and aesthetic atmosphere in healthcare architecture (Verderber, 2010; Verderber and Fine, 2000). A number of investigators have addressed, to varying degrees, the role of residentialist design amenities as therapeutic modalities in everyday RLTC settings. Rijnaard et al. (2016) cited 15 determinants within three superordinate themes that influence RLTC home residents’ sense of home: psychological factors, socialization, and built environment factors. Built environment factors consist of private space, semi-public space, personalization opportunities, assistive technology, ambiance, and the immediate outdoor environs. Numerous studies have addressed the role of residential-like architectural design in these settings (Bergland & Kirkevold, 2006; Calkins, 2009; Carr et al., 2013), in RLTC facility renovation processes (Neylon et al., 2019), and through the use of standardized metrics to help ascertain occupants’ design preferences most important to their success in engaging in activities of daily living (ADLs; Burton & Sheehan, 2010; Wahlroos et al., 2021; Wei & Li, 2021). In addition, the occurrence of loneliness among RLTC residents has been associated with specific physical environment design attributes (Nasrallah & Pati, 2021).

The transition from a RLTC setting to a 24/7 rehabilitation facility to a NIRS can result in physiologic functional decline, psychological depression, and social isolation unless adaptive design support interventions are structured and put in place *apriori* (Gromark & Andersson, 2020; Ngahama et al., 2016; Potter et al., 2018). In a study in Japan, Tsuchiya et al. (2019) found that unsafe, unpredictable residential settings undermine personal independence and well-being. In another Japanese study, conducted in 169 RLTC homes, the most effective plan configurations were identified based on “partitioning theory,” that is, facility and site context typology (Cao & Dewancker, 2021). In a North American investigation, Chaudhury et al. (2013, 2016) concluded that kitchen-dining areas in RLTC homes are a critical hub of social activity and should be accorded a high level of architectural design attention.

#### Personal space and cultural factors

A review of the Japanese evidence-based literature on environment and aging (Verderber & Song, 2005) identified room personalization and a respect for cultural and architectural traditions as core themes in preferred residential settings. Recently, proxemic spatial relationships in 168 RLTC settings in Japan was the subject of further research (Cao & Dewancker 2020; Cao et al., 2021). Five proxemic relationship types were identified as essential prerequisites in a successful floor plan configuration. A cross-cultural study that compared and contrasted Japanese and Western traditions centered on the resident’s bed-space zone in RLTC homes; Western cultural norms generally call for a larger amount of physical movement space around the bed (Yamaguchi, 2020). Residents have documented their own personal space from the standpoint of behavioral measures taken to self-personalize their room (van Hoof et al., 2015, 2016). The results were subsequently incorporated in administrative polices that guided spatial modifications to the study site facility. In a study of 632 residents of RLTC settings it was found that a residential ambience, social autonomy, control, and personal choice in RLTC unit design should be of high priority (Cater et al., 2021). In Canada’s Far North, the inclusion of decolonialist cultural traditions in First Nations communities was the subject of an investigation that yielded design prototypes for small-scale elder residences, for six, 12, and 18 residents; these were proposed as a policy alternative to the counterproductive historical and ongoing administrative practice of older persons in these indigenous communities being involuntarily relocated to impersonal, large-scale RLTC settings far from their ancestral community (Verderber et al., 2020).

### Nature and Landscape

#### Biophilia/therapeutic gardens

The therapeutic impact of outdoor green spaces and gardens in RLTC settings has been the subject of considerable research in recent years. The leading edge work of Claire Cooper Marcus (Cooper Marcus, 2007), and with Naomi Sachs, has framed this discourse in many respects; their use of the term *therapeutic gardens* describes what had previously been widely referred to as healing gardens (Cooper Marcus & Sachs, 2014). A therapeutic garden encompasses the properties and aesthetic features of a healing garden. However, the terminology of the latter has been problematic: is the garden itself healing? Chi et al. (2020) conducted a literature review on this general topic, identifying five principal themes conceptualizing person–nature transactions in healthcare-built environments: participatory design strategies, patterns of use-based transactions, frequency of engagement, impact assessment protocols, and health status outcomes. More specifically, blue (water) and green (vegetated) spaces have been differentiated with respect to their health status impacts (Finlay et al., 2015). Residents’ frequency of contact with gardens and related vegetated outdoor spaces has been examined (Eren et al., 2020; Hsish et al., 2021; Scott et al., 2022; Xie & Yuan, 2021), together with the impact of these spaces on cognitive restoration among residents (Cutler & Kane, 2005; Freeman et al., 2012; Ottosson & Grahn, 2006). A participatory design strategy consisting of residents being shown videos of landscape design options for an outdoor courtyard improvement project yielded end users’ design assessments (Yari et al., 2021). A related investigation consisted of an application of the Eden Alternative as a means to foster heightened person–nature transactions in RLTC outdoor settings based on a single-site case study in Chicago (Raske, 2010). Related, general guidelines were reported recently with respect to therapeutic biophilia amenities in SCUs for residents with dementia (Peters & Parekh, 2022).

Exterior RLTC site and campus amenities have been the focus of a number of recent investigations. Gonzalez and Kirkevold (2016) concluded that stable (hard surface) walkways, direct access to landmark features, visibility of outdoor spaces from indoors, the provision of seating, and appropriate prosthetic supports, that is, ramps, railing, foot lighting, and general lighting, are prerequisite in fostering more frequent usage. Design guidelines have been articulated for RLTC settings and their immediate outdoor environs based on person–nature precepts (Peters & Verderber, 2022; Rodiak & Schwarz, 2008); earlier work on this topic by Bengtsson and Carlsson (2006) had identified the importance of older users’ ability to frequent outdoor spaces without fear. In a study by Edwards et al. (2013), all 10 study participants experienced reduced agitation levels and the majority experienced reduced depression scores following the construction of an outdoor garden at their RLTC home. A similar reduction in agitation levels following exposure to outdoor garden spaces was identified by Dahlkvist et al. (2016). Design strategies for the greening of a geriatric hospital ward have also been examined. The introduction of plants and nature-themed wall surfaces reduced functional decline among older patients as assessed by geriatric unit staff (Van den Berg et al., 2021). The microclimate characteristics of outdoor spaces have been associated with positive therapeutic impacts on older users’ well-being in temperature-controlled conditions; study participants, however, were unable to discern when they were about to get too hot or too cold due to their reduced thermal sensitivity (Vecellio et al., 2021). Recent method-based research on this topic has centered on the use of semantic environmental descriptions of older persons and person–nature transactions (Bengtsson et al., 2015). Related research consisted of a seniors’ outdoor survey—as an observational tool for assessing RLTC outdoor environments containing 60 ratable items organized into five domains: access to nature, outdoor comfort/safety, walking and related activities, indoor–outdoor connections, and engagement with the world beyond (Rodiek et al., 2016).

#### Nature engagement and dementia

The role of nature and landscape with respect to persons with dementia has received increased attention across the years canvassed in this literature review. Calkins et al. (2007) found that such residents’ exposure to the outdoors resulted in modest improvements in sleep patterns, and a mixed or immeasurable impact on individual residents’ agitation levels under four conditions: winter/no activity, winter/inside activity summer/no activity, and summer/outside activity. The broad value of outdoor wandering gardens for persons with dementia has been reported by Chapman et al. (2007), Cohen-Mansfield (2007), Grant and Wineman (2007), Hernandez (2007), Whear et al. (2014), and Motealleh et al. (2019). A successful program to adapt outdoor gardening activities for persons with dementia was reported by Kwack et al. (2005). In a comparative study in the Netherlands, green care farms, traditional RLTC homes, and noninstitutional dwellings, 115 (Phase 1) and 100 additional settings (Phase 2) were observed and documented. Green care farm residents participated significantly more in domestic activities, outdoor/nature activities, and significantly less in passive nonnature activities compared to two other study cohorts (de Boer et al., 2017). A team led by Detweiler conducted two empirical studies on the therapeutic benefits of wandering gardens in RLTC homes, concluding that those who frequented these spaces more exhibited lower levels of agitation, with staff and family members concurring this affordance helps to suppresses inappropriate behaviors while concomitantly improving the quality of life for study participants (Detweiler, 2008). In a follow-up study Detweiler et al. (2009) found that scheduled medications, that is, antipsychotic prescription frequency, the incidence of physical falls, and fall severity scores decreased by 30% among older persons who most frequently used the facility’s outdoor wandering garden.

### Dementia Special Care Units (SCUs)

#### Immediate living spaces

A significant amount of evidence-based environment and aging research has been reported in recent years on the needs of institutionalized individuals with dementia on essential physical attributes of Special Care Units (SCUs)—increasingly referred to as *memory care units* within RLTC homes—and on associated policies pertaining to well-being, including the well-being and job performance of staff caregivers (Barrett et al. 2019; Molony, 2010). Policy reviews on this subject include those by van Hoof et al. (2009), who concluded that European countries with a high level of preexistent social support services for older persons tend to report successful outcomes in terms of resident and staff satisfaction and health status. Four key identified principles of eldercare human rights to be cognizant of are respect, freedom, dignity, and equality with regard to the planning and design of these care settings (Charras et al., 2016).

Comprehensive reviews of empirical person–environment research on residents with dementia in RLTC settings include the work of Chaudhury et al. (2014, 2018) and Marquardt et al. (2014). In part, they conclude that environmental cues need to be comprehensively attuned to these residents’ diminished cognitive abilities throughout the entire physical setting, that is, wayfinding signage, furnishings, lighting, color palettes, unit layout, and so on; socialization spaces are critical in this regard (Campo & Chaudhury, 2012). On the topic of wayfinding, Marguardt (2011) reviewed 169 research studies in SCU physical settings for dementia residents and distilled a set of design principles. Marquardt and Schmieg (2009) previously had distilled the critical design features of an effective wayfinding system. These reviews, together with Verbeek et al. (2010), concluded that small-scale units are most supportive of dementia residents’ ADLs with respect to kitchen/dining functions, personal autonomy, privacy, hygiene, socialization, and maintaining a meaningful degree of contact with nature. A review of the literature which focused on the central role of the RLTC kitchen-dining realm was reported by Chaudhury et al. (2013). In a related study in Japan, a core set of ADLs were identified: food pre/dining, sleeping, personal hygiene, dressing/undressing, praying, and educational/therapy activities (Yokoyama et al., 2009). Wong et al., (2014) and Tartarini et al. (2017) reported that nonmidrange ambient indoor temperature levels are associated with increased levels of resident agitation in SCUs. In the latter study, heightened agitation behavior was correlated with the number of hours residents were exposed to temperatures higher than 26 °C or in turn lower than 20 °C. Excessively high temperatures, low lighting levels and noisy social spaces resulted in a higher level of agitation (Garre-Olmo at al., 2012). In prior work related this topic, on thermal comfort and indoor air quality, van Hoof et al. (2010a, 2010b) reinforced these findings. Slaughter at al. (2007) field tested a set of prevalidated assessment scales to measure the efficacy of SCU physical and social attributes among residents with mid to late-stage dementia, while Olson and Albensi (2021) more recently have argued against the provision of excessively institutional settings which only serve to perpetuate the four countertherapeutic “A”s to be eschewed in RLTC physical settings: apathy, anxiety, agitation, and aggression.

#### Design interventions

SCUs and memory care units have been found to provide superior care for older persons with cognitive impairment and related physical disabilities compared to conventional, mainstream non-SCU RLTC settings, and SCUs are associated with fewer rehospitalization events, tube feeding, depression rates, agitated behaviors, and alternatively, greater personal autonomy, less environmental stress, and overall higher satisfaction (Cadigan et al., 2012; Calkins, 2018; Fisher et al., 2018; Fleming & Bennett, 2015; Orfield, 2015). Comparative studies of SCUs include comparing large, traditional units to small-scale nontraditional units (Afendulis et al., 2016;de Boer et al., 2021; De Rooij et al., 2012; Kane et al., 2007; Morgan-Brown et al., 2013). Small-scale open (deinstitutionalized) units, architecturally, were found to result in increased social interactions and satisfaction. Small-scale residential units of up to 15 beds, seating provided in open, visible circulation zones, a home-like ambiance, the presence of effective noise reduction measures, effective spatial transition zones from semi-public to private areas, adequate lighting, and a clearly visible staff workstation were associated with higher resident satisfaction (Milke at al., 2009; Verbeek et al., 2009). Design interventions inspired by Montessori principles, revolving around the primacy of the activity table, have been field tested in SCUs and memory care units with some success (Feng at al., 2018). Design considerations focused on the promotion of a residentialist sense of home in RLTCs have been reported by Eijkelenboom and Verbeek (2017) and Pollock and Fruggle (2013).

A neurological test battery and behavioral observation were data collection methods utilized in a study of 67 older persons who relocated from a traditional RLTC unit to a SCU. It was found that small-scale SCUs for dementia care are somewhat more supportive of cognitive spatial orientation needs (Kok et al., 2016). A theoretical model was put forth to describe physical, more abstract amenities preferred in SCUs based on two case studies in the United Kingdom. En suite bathrooms, small-scale residential clusters, and spaces with multiple use-affordances are of high priority (Burke & Veliz Reyes, 2021). Design precepts have been developed for dementia SCUs and memory care units to maximize these residents’ self-awareness in maximizing their remaining physical abilities, with respect to ADLs (Davis at al., 2009).

With regard to architectural design in support of successful wayfinding in SCU/memory care settings, residents have the greatest difficulty locating dining, social, personal hygiene spaces, and their own bedroom. Units designed for 7–10 residents are most recommended (Caspi, 2014). SCU residents who participated in an outdoor activity program exhibited maximum sleep duration patterns and less agitated behavior (Connell et al., 2007). Safety/security, private bedrooms, and adequate multisensory stimulation were the primary focus of design guidelines put forth by Fleming and Purandare (2010) based on review of 57 published research studies; provision of adjacent outdoor spaces was accorded somewhat less importance. Lee et al. (2016) studied staff caregivers, concluding that physical comfort, a familiar spatial context, and an organized, predictable physical setting contributes greatly to the well-being of residents. Finally, the impact of COVID-19 on the redesign (renovation) of SCUs and memory care units in the wake of the coronavirus pandemic has been investigated. The findings echo and reinforce the prepandemic consensus that small-scale units with all-private bedrooms, bathrooms, and social spaces that allow for adequate social distancing are most effective in combating the indoor transmission of infectious disease (Ferdous, 2021). The impacts of COVID-19 on older persons are further discussed here (below). Finally, Quirke (2018, 2021) examined and field tested the adaptation of mainstream postoccupancy assessment tools for specific application in RLTC dementia care settings. It was found certain existing tools and related metrics can be effectively redeployed to these facility contexts.

***With regard to architectural design in support of successful wayfinding in SCU/memory care settings, residents have the greatest difficulty locating dining, social, personal hygiene spaces, and their own bedroom***.

### Voluntary/Involuntary Relocation

The relocation experience for institutionalized older persons has been a source of controversy since the 1970s, stemming from research in the field of environmental gerontology that revealed the likely deleterious consequences of older persons’ involuntary relocation from NIRS contexts to nursing homes, and the impacts of inter-institutional nursing home relocation. Involuntary relocation from one architectural setting to another was then, and continues to be, of concern from both a policy and to a lessor extent built environment perspective. The role of the family in the relocation experience to a new RLTC facility has been the focus of recent evidence-based research. Why does this stream of research pertain to the role of the designed built environment? For one, exposure to a less environmentally supportive postmove facility setting can have a disruptive impact. Whether relocating from home to institution or between institutions, this type of change can be physically and emotionally debilitating. Further, postmove, the sudden loss of privacy and personal autonomy can be difficult to psychologically overcome or ameliorate, architecturally.

#### Relocation

Relocation generally consist of three phases: an anticipatory phase, the actual relocation itself, and the settling-in/adaptation (or non-adaptation) phase. In a policy review essay, de Boer et al. (2021) assert that further empirical research is warranted on the pre-relocation experience from the perspective of the resident, staff caregiver, and the family. Best practices to maximize the quality of life, postmove, and research on the impacts of a relocation to small-scale RLTC architectural settings is particularly needed. The meaning and significance of a supportive independent living physical setting prior to the point of relocation to an institution was addressed by Aminzadeh et al. (2010) and Wu and Rong (2020). Residents with dementia were the focus; it is essential that extra premove measures, facility-related and otherwise, are taken to ensure a successful relocation experience. Sudden transitioning from an assisted living facility to a RLTC SCU/memory care unit can be a source of consternation to family members; therefore, pre- and postmove family engagement is an essential component in this process. This can be accomplished by, in part, educating the SCU/memory care unit resident on the future physical setting where one will reside (Kelsey et al., 2009). The move from a RLTC facility to an acute care hospital has also been studied. In this regard, consistent premove medication protocols can help ensure a higher health status outcome in the relocated individual. Nursing interventions that involve, inform, and otherwise educationally prepare older persons prior to an inter-institutional relocation to a new, unfamiliar facility have been shown to inculcate in the individual a sense of unbroken perceptual control; this concomitantly helps to reduce adverse postmove outcomes after one has taken up residency in the new facility (LaMantia et al., 2010).

Social interactions and transactional strategies related to the built environment to reduce residents’ sense of loneliness and isolation, postmove, has been the subject of empirical research. Falk et al. (2011) reported of a relocated cohort that experienced a decrease in social connectedness in their new facility. In interviews, these subjects had viewed the premove preparatory phase as unpredictable and stressful. Multiple studies have concluded ill-planned relocations can result in multiple adverse outcomes for residents in the new, unfamiliar facility (Holder & Jollley, 2012; Jolley et al., 2011). In addition, the role of community infrastructure relative to the facility itself has been found to have an impact on prerelocated older persons. In a related study, in Australia, Williams et al. (2007) echoed this finding, advocating for a more meaningful role for local community health councils working in consort with RLTC home administrators during the prefacility closure phase, as this has frequently been a neglected area of national policy in Australia (and elsewhere). Similarly, the role of the continuing care retirement village has been examined from the perspective of relocation: Aging in place care models must be more compassionately attuned to residents’ changing functional abilities within the physical context and also within the local community; these measures are prerequisite to successful premove preparation to the new place of residence if even within the same campus setting (Cheek et al., 2006).

Involuntary relocation to a new, unfamiliar facility has been examined in the postmove phase from a clinical perspective (Capezuti et al., 2006). Relocation was found to be a stressful event, following the move itself. However, a move to a higher quality RLTC facility did not result in significant physical or mental health adverse outcomes. With respect to a voluntary move out of a conventional, mainstream RLTC facility to a small-scaled SCU/memory care unit within a larger RLTC facility, McFadden and Lunsman (2010) did not identify significant decline in socialization activity or related behaviors among the 22 study participants, all of whom had dementia. In a study conducted in Japan, it was concluded the community-at-large is a key stakeholder in an older person’s (successful) relocation from one’s private home or apartment to a RLTC setting. Desirable, accessible neighborhood amenities and their closeness to the new place of residence exerted a positive influence on well-being (Yamamoto, 2008).

A new, unfamiliar RLTC facility can adversely impact resident health status insofar as a higher incidence of falls, posttransfer, can occur among both those with or without a prior fall incident history (Castle, 2005). A statistically significant increase in fall incidents has been identified among posttransfer subjects (76.9%) compared to the pretransfer period (51.2%; *p* = .0001); 76% of those with a history of falling, premove, fell during the postmove period while 77.4% of subjects without a history of falls, premove, fell. Institutional relocation also can adversely impact cognitive performance, depression rates, and social engagement (although some pre- and postmove atrributional differences were nonsignificant, statistically). The study’s authors concluded more effort premove is necessary to acclimate the newly relocated to their new architectural setting. In a related 12-month study conducted in Canada, Hagen et al. (2005) concluded the administration of antipsychotic medications was significantly lower among a nonmove resident cohort, compared to a cohort that did relocate to a new RLTC facility. The physical setting itself was cited as one of many potential influences on this outcome.

In general, older persons tend to grow attached to their physical home base and, as a consequence, tend to respond poorly to being involuntarily uprooted. In post–Hurricane Katrina New Orleans, involuntarily relocated RLTC residents to a new RLTC facility experienced a higher rate of ulcers, postmove, and a higher rate of mortality (Engberg & Castle, 2008). However, it was not examined directly the extent to which the postmove facility, or the actual move, caused the reported adverse outcomes although the new facility was cited as a likely influencer. In a related study, Laughlin et al. (2021) found a significant decrease in attitude and mood/outlook, physical functionating, and cognitive performance in the relocated cohort, yet no significant increase in postmove mortality. In the realm of independent living and residential relocation, a study in Japan found that older person-study participants who were involved heavily in their premove planning phase, from an educational perspective, experienced a significantly higher level of satisfaction and well-being, postmove in their new residence. Inversely, reluctant residents, in the premove phase, experienced adverse outcomes in this regard (Yamada et al., 2014). It was found that among the premove “acceptance cohort,” the resident’s ability to self-personalize one’s own space at the new facility, postmove, had a positive impact on postmove resident satisfaction.

#### Family engagement

Relative to resident satisfaction and well-being in RLTC homes, the role of the family is increasing with respect to the built environment. More specifically, an emerging literature speaks to the growing role of the family in helping ease the transition to a new, unfamiliar RLTC facility. Family members are acting in a newfound role, in some cases, with respect to facility management policies. As to the function of family engagement, the inner profundities of the RLTC facility-to-hospital transfer decision-making process have been studied by Abrahamson et al. (2016). Based on semi-structured interviews with representatives of 20 families, the family was found to highly value a comfortable, attractive, home-like built environment. In a related investigation, family members as well as staff direct caregivers were the subject of a single-site case study in Australia in a 21-bed SCU/memory care unit (Cioffi et al., 2007). Study participants accorded high priority to a high-quality architectural environment as having a positive impact on residents’ quality of life, personal control, autonomy, a reduced level of agitation, improved sleeping patterns, and improved weight stability. Related to this, family members (and staff caregiver) study participants have identified excessive noise as a key stressor that can adversely impact residents’ well-being (Garcia et al., 2012, see below). On the subject of wayfinding and stress in RLTC homes, a study conducted in Northern Ireland and Scotland was conducted consisting of a sample of 40 family representatives, 29 residents who experienced dementia, and 11 direct caregivers (Innes et al., 2011). Families and caregivers alike cited effective wayfinding cues and outdoor therapeutic wandering gardens as a high priority in residents’ well-being. The adverse impact on both families and residents during facility lockdowns has been addressed in the context of the COVID-19 pandemic. Gaugler and Mitchell (2021) recently examined this issue, concluding that RLTC facilities in the future need to be newly built and/or renovated to allow family members to enter the new facility to interact to the extent medically allowable directly with the resident. This includes, in facility lockdown situations, spatially neutral “safe zones” where family member and resident can safely interact without compromising one another’s health status.

### Infection Control/COVID-19/Environmental Stress

Numerous quantitative and qualitative studies have examined the functions of infection control, environmental stress, and most recently, the impact of COVID-19 in RLTC built environments. This work has centered on indoor air quality, infection transmissibility, ventilation best practices and its health impacts, excessive noise, light and illumination therapy in regulating circadian rhythms and sleep patterns in controlling depression and agitation behaviors in residents, and the role of health policy.

#### Ambient conditions, safety, and infection control

The literature on non-COVID-19-related infection control is substantial. The installation of pulsed-xenon ultraviolent room disinfection devices led to a decrease in RLTC home microbial infection and hospitalization rates. Exposed surfaces sanitized vis-à-vis this method proved superior to manual cleaning to decrease infectious microbes (Kovach et al., 2017). In a qualitative investigation, Stone et al. (2015) advocated for greater awareness of a range of preventative measures to mitigate the occurrence of infections in these settings. Exposure to chemical-based indoor air pollutants adversely affected health status; this adverse effect increased with residents’ age and particularly in poorly ventilated conditions (Bentayeb at al., 2015).

The impact of lighting types and light therapy has been the subject of numerous investigations. These studies include the impact of ambient bright light on residents’ diurnal sleep patterns, agitation levels, rates of depression, and sleep-wake activities. In a study of agitation behaviors, Barrick et al. (2010) concluded that bright light exposure is ineffective in reducing agitation in persons with dementia and may in fact exacerbate agitation behaviors. Dowling et al. (2005) reported that persons with dementia did not exhibit significantly more stable rest-activity rhythms over a 10-week observational period, compared to a control group not exposed to 1 hr of bright light in either morning or afternoon sessions. Giggins et al. (2019) concluded that only some study participants responded positively to a bright light intervention session, subsequently exhibiting heightened activity levels, reduced-length periods of daytime sleep, and heightened mood, with the majority exhibiting no significant improvement in these respects. As for the function of gender-based differences in light therapy research in RLTC settings, Hickman et al. (2007) found that positive health status outcomes were most pronounced for women, particularly during morning-hours light therapy sessions. However, this same pattern was not identified among male residents.

De Lepeleire et al. (2007) studied illumination levels in eight RLTC homes in Belgium, concluding that at dusk and during evening hours the lighting was inadequate and contributed to increased fall incidents among residents at these specific times. Kim et al. (2021) found the lighting levels recorded in two U.S. homes, as assessed by 57 older person-participants, as consistently below recommended industry-wide lighting standards. In a randomized control trial, bright light therapy was found to have a modest positive impact on subjects’ overall cognitive functioning (Riemersma et al., 2008). Fetviet and Bjorvatn (2005) concluded that bright light’s alerting effects as a treatment protocol resulted in the majority of dementia subjects’ reduced daytime length of sleeping from rising time to 3:00 pm during the study period. Sloane et al. (2007) reached a similar conclusion regarding a measurable positive impact of bright light therapy sessions in the morning, with ambient daylight more effective than stationary artificial light sources. This was also corroborated by Royer et al. (2012), who exposed subjects to blue light, and a control group, to red light. Similarly, Friedman et al. (2012) and White et al. (2013) concluded this treatment modality did result in residents’ reduced periods of daytime sleeping, less insomnia at night, and a decrease in depression level. In the latter study, a 24-hr automated algorithm was pretested to control lightness/darkness levels.

As for the impact of excessive noise levels on stress, Joose (2012) and Garcia (2012) concluded that noise and associated spatial–physical attributes have an adverse impact on RLTC residents’ agitation behaviors. However, prenoise exposure agitation may be key in predicting this outcome (Joose, 2011). In a review of the literature on this topic, Thomas et al. (2020) found that international standards are currently in flux and place-specific variables should be taken into consideration when implementing noise abatement policies. As for the influence of indoor air temperature, Tartarini et al. (2017) reported that temperature levels beyond the comfort range of 20–26 °C caused agitation, and recommended temperature fluctuations should be minimized for dementia residents in RLTC settings. On the topic of fall occurrences and resident safety, while a substantial literature exists on the prevalence and impact of falls among the institutionalized aged, only one study was reported in recent years. Jiang et al. (2021) found that a mix of immobile and ambient environmental hazards increase the likelihood of fall incidents. The most common hazards are inadequate/nonexistent handrails, unnavigable floor surfaces, and inadequate illumination. Indirectly related, Yasuda and Miura (2021) examined the type and prevalence of visual blind spots, concluding the elimination of such physical barriers can improve residents’ perceived sense of well-being but must be balanced with the resident’s privacy needs.

#### COVID-19

The coronavirus pandemic (2020 to present) gave rise to a growing literature on its impacts in RLTC environments. A nursing home crowding index was created and tested in the context of COVID-19 infection and mortality rates in Canada (K. A. Brown et al., 2021). The major finding was that overcrowded conditions were a common occurrence; these settings were significantly more prone to experiencing larger and deadlier disease outbreaks. In a design-centric review of the impacts of COVID-19 in RLTC settings, Anderson et al. (2020) concluded the role of architectural design in the residential unit milieu had a profound impact on residents’ infection and mortality rates throughout the pandemic. They stressed the importance of compassionate, health-promoting design, and presented a recommended unit layout featuring all private bedrooms, private hygiene facilities, appropriately resilient transition/circulation zones to facilitate personal distancing, therapeutic gardens in close proximity, informal, close-by staff workstations, health-promoting (including natural) ventilation systems, ultraviolet lighting, and a home-like, small-scaled unit configuration.

Lynch and Goring (2020) recommend a series of measures to mitigate infectious disease so negative air pressurization. Thompson et al. (2020) and Olson et al. (2021) adopted an international perspective, echoing Anderson et al. (2020) in advocating for small-scale clustered residential units with all private bedrooms/bathrooms, the ability to isolate infected residents to achieve transmissibility reduction, and updated guidelines for the design and construction of these facilities. This call for updated regulations and standards for disease and infection control was underscored by Z. Wang (2021), who addressed the situation in China’s COVID-19 emergency hospitals rapidly erected in Wuhan and elsewhere in the country in early 2020. In a review, Zhu et al. (2022) underscored the importance of increased privacy, personal autonomy, and interpersonal distancing RLTC architectural minimum design standards.

### Sustainability/Facility Management

In a case study, four administrators and four architects specializing in RLTC facilities collectively developed three prototype floor plans, consisting of a traditional shared bedroom (semi-private) layout; an enlarged semi-private bedroom layout; and an all-private bedroom layout. It was concluded the all-private room unit configuration was most cost-effective and concomitantly promotes the most advantageous health outcomes (Calkins & Cassella, 2007). This finding corroborates the related studies reported above but was the first to focus on the long-term cost-effectiveness of all-private room homes, and the increasingly accepted view that semi-private bedrooms are unsafe from a disease and infection control perspective because they violate residents’ self-dignity, privacy and personal autonomy needs, and prohibit sufficient personalization.

Sustainable energy operations in these facilities have also been the focus of recent research. This includes usage of heat and hot water systems in RLTC homes, together with measures to further conserve annual energy consumption and operational costs (Ivanko et al., 2020), thermal resistance of building materials in RLTC facilities (Sun et al., 2020), and energy efficient renovation measures (Teni et al., 2019). In an investigation on the relationship between LEED and the presence/absence of salutogenic and biophilic design features in RLTC settings, a cross section of 18 LEED facilities in the United States and Canada were analyzed (Verderber & Peters, 2019). It was found LEED criteria alone do not necessarily ensure a LEED certified facility will exhibit salient any significant salutogenic and/or biophilic design affordances for its inhabitants. The highest composite-scored case studies did exhibit, however, ecologically based design features as well as salutogenic/biophilia features.

Finally, the role and function of disaster preparedness planning and protocols in RLTC built environments was the subject of research by Peterson et al. (2014). Among the findings, five resilience measures are called for (1) cooperatively integrating these homes with the external agency-entities on which they depend; (2) assessing risks and available resources using knowledge of the external and internal environment arrived at in consort with external support entities; (3) anticipating problems based on thorough risk assessment and establishing appropriate action-based response plans; (4) testing these plans vis-à-vis routine exercises and drills to specifically address unanticipated vulnerabilities and infrastructural impediments; and (5) evaluating postoccupancy facility performance to continually upgrade the level of facility resilience and community-based infrastructural support.

### Design Trends/Prognostications

Recent design trends in RLTC built environments largely focus on the health promoting aspects of all-private rooms, a *residentialist* home-like architectural aesthetic and ambiance, the presence of person–nature connections allowing residents and others to directly and meaningfully engage the exterior realm, and the growing role of family members in the planning, design, and day-to-day life of these care settings. The role of staff caregivers is similarly evolving with respect to the deinstitutionalization of their workspaces, and amenities provided for staff personnel as a means to heighten recruitment, retention, morale, outlook, and productivity.

#### Green house model (GHM)

The GHM has arisen in recent years as an increasingly popular architectural alternative to the traditional 24/7 *nursing home* RLTC facility. Its origins date to 2001 as an initiative of the Robert Wood Johnson Foundation to counter the sheer institutionalism of the conventional nursing home (Robert Wood Johnson Foundation, 2022). A GHM home is fundamentally *residentialist*. It is a small-scale alternative, where all food is prepared on-premises, medical equipment is present yet not openly visible, and staffing patterns differ from conventional RLTC facilities. At this writing more than 260 GHM facilities in 32 states in the United States are open or in so the underlying premise of the large-scale institution, its semi-private bedrooms, and often-communal hygiene facilities. In so doing, it thoroughly rejects the hospital-like legacy of geriatric facilities of the past (Kane et al., 2007). Numerous quantitative and qualitative investigations of this recent building type have been reported. Afendulis et al. (2016) reported that adoption of the GHM led to a reduced rate of rehospitalization of RLTC home residents without sacrificing the quality of clinical care or other aspects of everyday life. The majority of study participants also experienced less usage of catheters and incurred significantly fewer pressure ulcers.

Staff performance and psychological well-being have been studied relative to the type of RLTC setting where one is employed. P. B. Brown et al. (2016) reported that staff who work in GHM settings experience greater job longevity and higher job satisfaction compared to a control group comprised of staff who work in a traditional (non-GHM) facility. Similar positive benefits have been reported with residents as well as family members (Fishman et al., 2016), specifically, with respect to adjacent landscaped outdoor spaces, the small scale (bed capacity) of the unit, and all-private bedrooms. In postoccupancy evaluations of the first four GHM facilities, Cutler and Kane (2009) reported it a viable and progressive alternative to the conventional nursing home. Among the findings, residents spend more time in their private bedrooms in a variety of activities including hosting visitors, often with the door closed versus always being on view in centralized “public” social activity areas. Cohen et al. (2016), in a study of end-user direct participation in the facility design process, concluded, in part, more effort is warranted to solicit end-user input throughout the design phase of new GHM facilities.

#### Trends/prognostications

The aging of societies around the world is resulting in ever-burgeoning numbers of old-old, that is, persons older than age 85. Environmental gerontology, as a discipline, must be solution-driven in response (Schwarz, 2012; C. Wang & Kuo, 2006). An essay by Craig (2017) envisions a future where thousands of new RLTC settings will be needed to house the expanding ranks of the aged globally. Using a case study method in an international context, Kerbler (2016) concluded, in part, that society is yet to fully accept the urgency of the need to rapidly provide more housing alternatives for the aged. Regnier (2018) reported on field research conducted in the United States, the Netherlands, Sweden, and Denmark on alternative assisted living and transitional architectural environments for increasingly cognitively and physically frail individuals. A related study in the Netherlands addressed the utility of cognitive mind mapping in ascertaining resident and other stakeholders’ cognitive interpretations of their “ideal” physical setting (van Hoof et al., 2014) Engelen et al (2022), in a review of sixty-five published studies and reports, identified seven themes on the relationship between health status, facility design, and quality of life: biophilia, indoor environmental quality, assistive technology, wayfinding, socialization affordances, with limited empirical attention reported relaive to safety/security or adaptable design amenties.

Virtual reality assistive technology was the subject of a study in Sweden where RLTC home residents viewed simulated nature: scenes of nature and landscape were identified as a source of positive stimulation and distraction from residents’ everyday routines (Lundstedt et al., 2021). With respect to a non-VR study of mental health, the physical design of these settings has been examined as a means to counter the rise in loneliness that unprecedented numbers of older persons will experience as they live ever-longer lives and inadvertently lose meaningful longstanding personal relationships. Symbolic interactionism, and affordance theory, were among the theoretical perspectives employed to examine this phenomenon (Nasrallah & Pati, 2021). Additionally, this demographic trend will directly impact the future education of design professionals (Orfield, 2013), with the aged being a source of much potential new knowledge for incorporation in the architectural and landscape design professions (Pirinen, 2016). Finally, an excellent, comprehensive report was issued in 2022 in the United States by the *National Academies of Science, Engineering & Medicine* on the present and future of aging in contemporary American society. An entire chapter was devoted to current and future trends in the design of built environments for older persons. Included was a literature review on best practices and as such reviewed many of the sources cited in the present review. Among the conclusions, the GHM is viewed as a progressive, best practice strategy, and the pronounced trend toward the all-private bedroom RLTC unit is advocated as the new baseline industry standard.

Each citation reported in Table 1 was then assessed and further categorized according to its thematic type as a function of its key focus area(s). This process is reported in Table 2. In this table, the eight thematic content areas are displayed in relation to resident outcomes, those which address staff (and family engagement) outcomes or both types of outcomes. Quantitative studies are distinguished from qualitative studies and policy/theory essays. In many cases, a given citation addresses multiple key foci, such as resident satisfaction as well as health status, as well as staff performance. In these cases, the total number of issues addressed in a given citation—and across all citations within its thematic content area—is reported in Table 2; this is done for resident and for staff/family engagement outcomes. Restated, in many cases a given study, policy review or theory essay addressed multiple issues and multiple user constituencies. The far-right column in Table 2 reports the actual number of citations reported in Table 1 on a theme-by-theme basis. This process yielded a pattern that clearly shows the resident is a primary focus of every one of the 202 citations to some degree. Secondly, the caregiver staff is a secondary focus of concern a cross the compendium of citations, while the issue of family engagement, comparatively, is a tertiary focus of concern. A closer perusal of Table 1 together with Table 2 will yield further insight as to which trends became more pronounced in the literature across the 17 years covered by this review based on citations’ date of publication. Space limitations do not allow for a further analysis of these data here from this standpoint. However, the reader is invited to peruse Table 1 as well as Table 2 for a deeper insight into this aspect of this multifaceted body of literature.

**Table 2.** Residential Built Environments for Older Persons (2005–2022): Quantitative Investigations, Qualitative Investigations, and Essays—Content Areas 1–8.

Thematic Content Areas 1–8	Resident Outcomes				Non-Resident Outcomes				
	Quality of Life	Health Status	Infection Control	NC^a^	Staff Satisfaction/Performance	Family Involvement^b^	Facility-Based Policy Input	NC^c^	Total NC^d^
1. Community-based non-RLTC settings									
Immediate neighborhood and urban environment	4^e^/4^f^	—	—	8	—	—	—	—	8
Residing in one’s existing home	5/7	—	—	12	—	—	—	—	12
Multigenerational dwelling strategies	5/5	—	—	10	—	—	—	—	10
2. Residentalism									
Design considerations and case studies	5/8	—	—	15	1/–	—	—	1	15
Personal space and cultural factors	4/4	1/–	—	9	—	—	—	—	8
3. Nature and landscape									
Biophilia/therapeutic gardens	16/5	4/1	—	26	4/1	—	–/2	7	21
Personal space and cultural factors	9/5	4/–	—	18	2/–	1/–	—	3	14
4. Dementia Special Care Units (SCUs)									
l Immediate living spaces	7/9	3/–	1/–	20	3/1	–/3	–/1	8	20
Design interventions	14/7	7/2	1/–	31	–/3	–/1	1/4	9	21
5. Voluntary/Involuntary relocation	12/8	11/2	—	33	1/2	2/5	2/6	18	21
6. Infection control/COVID-19/environmental stress									
Ambient conditions, safety, and infection control	14/1	22/–	9/1	47	2/2	1/–	5/1	11	22
COVID-19	6/1	7/–	7/–	21	—	—	–/1	1	7
7. Sustainability/facility management	–/3	3/–	–/2	8	—	—	3/3	6	6
8. Design trends/prognostications									
Green house model	3/3	3/–	–/1	12	1/4	2/–	3/3	13	6
Trends and prognostications	6/5	3/–	2/–	16	–/6	–/5	3/8	22	11
									204

^a^ Total number of resident outcome citations reported by category. ^b^ Citation addressing family role and facility policies. ^c^ Total number of nonresident outcomes by thematic category. ^d^ Total number of citations reported by thematic category Table 1. ^e^ Quantitative-based methodology. ^f^ Qualitative-based methodology/review/theory essay.

### Summary and Conclusions

This comprehensive literature review has underscored the importance of noninstitutional, community-based residential supports, walkable neighborhoods and retail and civic amenities in close proximity. Also, transit connectivity, establishing a genuine sense of place, self-empowering territorial imperatives, and attention to cross-cultural considerations. Also of importance is the therapeutic role of nature, safe wandering gardens, and person–nature engagement opportunities, particularly for older persons with dementia and related forms of cognitive impairment. More specifically, the planning and design of a SCU/memory care unit as part of an RLTC home (or as an autonomous facility) calls for innovative design—in light of the growing demand for this type of care unit. Also of priority is the issue of institutional relocation, including pre- and postmove impacts on resident well-being, mortality, staff well-being, and job performance and the role of family members with respect to the built environment. Multigenerational independent living was also identified as an area that has garnered increasing qualitative and quantitative research attention since 2005.

This review also addressed health status critical issues: disease and infection control, patient safety, privacy, personal autonomy, dignity, personal distancing, and the adverse impact of COVID-19 in RLTC built environments. Also reviewed was the role and adverse impacts of environment-based stressors including excessive noise, countertherapeutic lighting, spatial and aesthetic minimalism, poor indoor air quality, overcrowding, lack of meaningful engagement with the exterior realm (nature/landscape), and inflexible, nonadaptable interior living spaces or those difficult to spatially navigate. These conditions are tantamount to banal institutionalism. Also reviewed were ecological, cost-containment, and facility management best practices. Finally, recent trends, including the popular GHM, were reviewed, along with anticipated trends and prognostications for the future of both institutional and noninstitutional long-term care-built environments. This comprehensive review was inspired by a recent report whose aim was to reassess the planning and design of environments for older persons in the context of the adverse impacts of COVID-19 (Verderber, 2022).

The major conclusions of the present review are as follows:

1. *The Deleterious Impacts of the Coronavirus Pandemic*—The COVID-19 pandemic revealed the failures of many 24/7 long-term care residential facilities as perilous places to live or work. Overcrowded conditions and the lack of personal distancing space in bedrooms and in social activity areas can foster unacceptably high rates of viral transmission. The need for infection control must be balanced with a home-like setting that affords individual choice and personal autonomy. The evidence-based literature calls for a noninstitutional *residentialist* architectural aesthetic balanced with a high degree of infection control measures. Many RLTCs currently in operation were constructed without strict infection control measures foremost in priority and yet a balance between this and a residentialist aesthetic is highly recommended. Concurrently, a reappraisal of minimum facility planning and design standards is warranted—without sacrificing the qualities of a home-like, noninstitutional setting.
2. *The Primacy of Personal and Spatial Autonomy*—The literature speaks to the need for personal privacy and spatial autonomy within the RLTC milieu. Numerous studies call for all-private bedrooms housed within distinctly identifiable units, allowing direct access to the outdoors, with significantly lower per-unit bed capacities compared to the past. Multiple studies recommend RLTC units of typically no more than 12 beds. The impact of the movement toward all-private bedrooms has most directly manifested in Green House RLTC settings constructed in the past decade. This movement continues to grow in popularity and is currently bifurcating, to some degree, although the core premise remains constant—smaller is better, personal privacy, autonomy, and a medically safe physical setting are a right for everyone, not a privilege for only the few. As previously stated, a number of studies address the criticality of balancing these concerns with infection control, without operational standards occluding the provision and daily maintenance of an inviting residential atmosphere and aesthetic.
3. *Increased Attention to the Amelioration of Environmental Stressors*—A distinct stream of research has been reported in the peer-reviewed literature since 2005 on the impact of various environmental variables and their corresponding adverse impacts on well-being and health status in RLTC homes. While this literature remains inconclusive, suffice to say, underexposure at one extreme, and overexposure, on the other, can result in deleterious outcomes. The variables surveyed in this regard include the impact of light therapy sessions on the modification of residents’ circadian rhythms, sleep patterns, and agitation behaviors. Second, the impact of healthful ventilation systems—both natural and mechanical—in RLTC homes is currently being examined to an unprecedented degree due to COVID-19. Third, numerous studies point to the therapeutic benefits of the resident being able to spend increased time outdoors. A number of studies support the affordances of wandering gardens and related vegetated exterior spaces that allow the resident to commune directly with nature and landscape.
4. *The Growing Acceptance of Residents’ Support Infrastructure*—The role of participatory decision-making in the RLTC milieu continues to evolve. The involvement of the resident and the resident’s family has been shown to have a positive impact related to facility choice, usage, the therapeutic use of interior and outdoor spaces, renovation and related physical environment improvement initiatives, and in daily facility management policies. In addition, the role of multigenerational built environments has been receiving increasing research attention in everyday noninstitutional aging in place settings at home, and in the design of RLTC settings where an attempt is made to embed the RLTC facility within or near normative residential neighborhoods in the immediate broader community. As for the plight of the direct caregiver, the COVID-19 pandemic has been especially challenging for frontline nurses and other staff who witnessed firsthand such widespread suffering and death (T. Brown, 2021).
5. *Confluence with Broader Healthcare Research-Design Trends and Typologies*—The diversity of issues identified in this 2005–2022 review paralleled a number of broader themes and empirical research results that have already been accepted as “mainstream” by healthcare facility planners and designers beyond the noninstitutional and RLTC milieus per se. In the past, the nursing home was considered a building type onto itself. It was, too often, little more than a mini-hospital. Their minimalist interiors and lack of amenities—conditions especially problematic in high rise nursing homes, offered little in the way of genuine residential living supports. Aspects of this review that paralleled, mirrored, broader trends across the spectrum of building types for health/healthcare include an increasing focus on the therapeutic affordances of nature and landscape, the health status impact of proper lighting and ventilation, acoustical privacy, the increasing importance of ecological site planning and facility design, and the increasing role of RLTC home disaster preparedness in light of the unfolding climate crisis and its implications for older persons everywhere.

## Discussion

This review has endeavored to answer two fundamental research questions: What significant trends are discernable in recent research (2005–2022) on the role of the residential built environment in the lives of older persons? Second, in what ways has the coronavirus pandemic impacted the use and design of residential settings for older persons, and what specific recommended design interventions have emerged both for current reappraisal and for the future? In terms of under-addressed topics, scant attention has been devoted to the role of assistive technologies, that is, smart house digital technologies, robotics, and the role of artificial intelligence, in environments for older persons. The role of virtual reality also warrants more attention in this regard. Third, design prototyping continues to lack research attention in comparison to the extensive use of mock-ups and other means to elicit direct user input in the RLTC planning and design process. Similarly, insufficient attention has been devoted to successful case studies on the retrofitting of home-based aging in place residential settings. Fourth, too little attention has been devoted to the potentialities of multigenerationality and the potential of mixed-use 24/7 campuses, as well as the provision of nearby accessory housing for families, proximity to recreational, retail, community centers, arts organizations, and related civic and educational facilities. Numerous examples of these recent trends, nevertheless, can be found variously on European RLTC campuses in urban, suburban, exurban, and in rural settings. Case studies such as these are, unfortunately, seldom exposed to the rigor of a thorough postoccupancy performance assessment. Tangentially related has been the lack of a distinct research literature on the therapeutic benefits of art in the RLTC milieu, with no evidence-based research on this topic published since 2005.

Relatively little peer-reviewed research has been published on ecological sustainability in RLTC settings with the few publications on this topic scattered and uncomprehensive. It is hoped future evidence-based research will address this issue, together with further inquiry into the therapeutic role of salutogenic and biophilia-inspired design. Suffice to say, the intensifying global climate crisis calls for eco-humanist paradigms that will benefit the everyday life of older persons in residential settings, institutional or otherwise (Verderber & Peters, 2017). Correspondingly, pandemic-related built environment considerations in residential environments for older persons warrant increased research and design attention. Cross-cultural, interdisciplinary collaborations are needed in order to more effectively coalesce the expertise of health policy experts, direct care providers, researchers, and the many specialists who plan, design, and construct these built environments.

***Relatively little peer-reviewed research has been published on ecological sustainability in RLTC settings with the few publications on this topic scattered and uncomprehensive. It is hoped future evidence-based research will address this issue, together with further inquiry into the therapeutic role of salutogenic and biophilia-inspired design***.

The field of environment and aging continues to rapidly expand and evolve. As we age, our built environment needs dramatically change. The unmet need in terms of age-appropriate housing, healthcare facilities, and related community infrastructural amenities for older persons will continue to ever-increase unless proactive, ameliorative measures are taken. Evidence-based research, and therapeutic environmental and architectural built environments for older persons, are now more important than perhaps ever due to the sheer scale of the challenge to provide eco-humanist-built environments that conserve finite nonrenewable natural resources. Architects, landscape architects, interior designers, artists, lighting, and equipment specialists have much expertise and insight to offer. Core design considerations must strive to mitigate and ultimately eradicate adverse medical outcomes without dismissing the Vitruvian precepts that speak to the overarching need for architecture to provide commodity, firmness, and delight.

## Implications for Practice

- Evidence-based research and design are reported in the 2005–2022 period on the state of the art in NIRS for older persons and also RLTC built environments for older persons.
- This knowledge base has direct implications for site and facility planning, design, facility management, and postoccupancy performance assessment with respect to residential environments for older persons.
- The need for home-retrofitting is pronounced, as is multigenerational housing, as these supports are critical to older persons living independently. In the 24/7 RLTC milieu, a major shift is underway to provide smaller scale all-private room residential units housing clusters of 12–15 residents per “house” with all-private bath/shower rooms.
- The COVID-19 pandemic placed unprecedented focus on the need for personal distancing in residential environments for older persons to minimize infectious disease transmission. However, this is best accommodated by not over-isolating residents from one another and thereby precluding essential social transactions necessary to counter loneliness and depression.
- The deleterious impact of environmental stressors, that is, excessive light, noise, overcrowding, and the absence of meaningful, sustained engagement with landscape and nature emerged as thematic areas of concern.
- Finally, prognostications for the future include the acceleration of innovative architectural advancements in the provision of supportive, compassionate built environments for older persons globally.
